# Pathophysiology of chikungunya virus infection associated with fatal outcomes

**DOI:** 10.1016/j.chom.2024.02.011

**Published:** 2024-04-10

**Authors:** William M. de Souza, Marcilio J. Fumagalli, Shirlene T.S. de Lima, Pierina L. Parise, Deyse C.M. Carvalho, Cristian Hernandez, Ronaldo de Jesus, Jeany Delafiori, Darlan S. Candido, Victor C. Carregari, Stefanie P. Muraro, Gabriela F. Souza, Leda M. Simões Mello, Ingra M. Claro, Yamilka Díaz, Rodrigo B. Kato, Lucas N. Trentin, Clauber H.S. Costa, Ana Carolina B.M. Maximo, Karene F. Cavalcante, Tayna S. Fiuza, Vânia A.F. Viana, Maria Elisabeth L. Melo, Clarissa P.M. Ferraz, Débora B. Silva, Larissa M.F. Duarte, Priscilla P. Barbosa, Mariene R. Amorim, Carla C. Judice, Daniel A. Toledo-Teixeira, Mariana S. Ramundo, Patricia V. Aguilar, Emerson L.L. Araújo, Fabio T.M. Costa, Thiago Cerqueira-Silva, Ricardo Khouri, Viviane S. Boaventura, Luiz Tadeu M. Figueiredo, Rong Fang, Brechla Moreno, Sandra López-Vergès, Liana Perdigão Mello, Munir S. Skaf, Rodrigo R. Catharino, Fabiana Granja, Daniel Martins-de-Souza, Jessica A. Plante, Kenneth S. Plante, Ester C. Sabino, Michael S. Diamond, Eliseo Eugenin, José Luiz Proença-Módena, Nuno R. Faria, Scott C. Weaver

**Affiliations:** 1 Department of Microbiology, Immunology and Molecular Genetics, University of Kentucky, College of Medicine, Lexington, KY, USA; 2 Department of Microbiology and Immunology, University of Texas Medical Branch, Galveston, TX, USA; 3 World Reference Center for Emerging Viruses and Arboviruses, University of Texas Medical Branch, Galveston, TX, USA; 4 Global Virus Network, Baltimore, MD, USA; 5 Laboratory of Molecular Immunology, The Rockefeller University, New York, NY, USA; 6 Laboratório Central de Saúde Pública do Ceará, Fortaleza, Ceará, Brazil; 7 Laboratory of Emerging Viruses, Department of Genetics, Microbiology and Immunology, Institute of Biology, University of Campinas, Campinas, São Paulo, Brazil; 8 Department of Pathology, University of Texas Medical Branch, Galveston, TX, USA; 9 Laboratory of Immunobiotechnology, Biotechnology Center, Federal University of Paraíba, João Pessoa, Paraíba, Brazil; 10 Department of Neurobiology, University of Texas Medical Branch, Galveston, TX, USA; 11 Coordenação Geral dos Laboratórios de Saúde Pública, Secretaria de Vigilância em Saúde, Ministério da Saúde, Brasília, Brazil; 12 Instituto de Ciências Biológicas, Universidade Federal de Minas Gerais, Belo Horizonte, Minas Gerais, Brazil; 13 Innovare Biomarkers Laboratory, School of Pharmaceutical Sciences, University of Campinas, Campinas, São Paulo, Brazil; 14 MRC Centre for Global Infectious Disease Analysis, Department of Infectious Disease Epidemiology, School of Public Health, Imperial College London, London, UK; 15 Department of Zoology, University of Oxford, Oxford, UK; 16 Instituto de Medicina Tropical, Faculdade de Medicina da Universidade de São Paulo, São Paulo, Brazil; 17 Department of Biochemistry and Tissue Biology, Institute of Biology, University of Campinas, Campinas, São Paulo, Brazil; 18 Departamento de Moléstias Infecciosas e Parasitárias, Faculdade de Medicina da Universidade de São Paulo, São Paulo, Brazil; 19 Department of Research in Virology and Biotechnology, Gorgas Memorial Institute of Health Studies, Panama, Panama; 20 Institute of Chemistry and Center for Computing in Engineering and Sciences, University of Campinas, Campinas, São Paulo, Brazil; 21 Programa de Pós Graduação em Bioinformática, Instituto Metrópole Digital, Universidade Federal do Rio Grande do Norte, Natal, Rio Grande do Norte, Brazil; 22 Laboratory of Tropical Diseases, Institute of Biology, University of Campinas, Campinas, São Paulo, Brazil; 23 Center for Tropical Diseases, Institute for Human Infection and Immunity, University of Texas Medical Branch, Galveston, TX, USA; 24 Coordenação Geral de Atenção às Doenças Transmissíveis na Atenção Primária, Departamento de Gestão ao cuidado Integral, Secretaria de Atenção Primária à Saúde, Ministério da Saúde, Brasília, Brazil; 25 Universidade Federal da Bahia, Faculdade de Medicina, Salvador, Bahia, Brazil; 26 Fundação Oswaldo Cruz, Instituto Gonçalo Muniz, Laboratório de Medicina e Saúde Pública de Precisão, Salvador, Bahia, Brazil; 27 Hospital Santa Izabel, Santa Casa de Misericórdia da Bahia, Serviço de Otorrinolaringologia, Salvador, Bahia, Brazil; 28 Virology Research Centre, Ribeirão Preto Medical School, University of São Paulo, Ribeirão Preto, São Paulo, Brazil; 29 Sistema Nacional de Investigación from SENACYT, Panama, Panama; 30 Biodiversity Research Centre, Federal University of Roraima, Boa Vista, Roraima, Brazil; 31 D’Or Institute for Research and Education, São Paulo, São Paulo, Brazil; 32 Experimental Medicine Research Cluster, University of Campinas, Campinas, São Paulo, Brazil; 33 Departments of Medicine, Molecular Microbiology, Pathology & Immunology, Washington University School of Medicine, Saint Louis, MO, USA; 34 Institute for Human Infection and Immunity, University of Texas Medical Branch, Galveston, TX, USA

**Keywords:** chikungunya virus, chikungunya death, arbovirus, mosquito-borne virus, alphavirus, pathophysiology, inflammation, hemodynamic disorders, central nervous system infection

## Abstract

Chikungunya virus (CHIKV) is a mosquito-borne alphavirus that causes acute, subacute, and chronic human arthritogenic diseases and, in rare instances, can lead to neurological complications and death. Here, we combined epidemiological, virological, histopathological, cytokine, molecular dynamics, metabolomic, proteomic, and genomic analyses to investigate viral and host factors that contribute to chikungunya-associated (CHIK) death. Our results indicate that CHIK deaths are associated with multi-organ infection, central nervous system damage, and elevated serum levels of pro-inflammatory cytokines and chemokines compared with survivors. The histopathologic, metabolite, and proteomic signatures of CHIK deaths reveal hemodynamic disorders and dysregulated immune responses. The CHIKV East-Central-South-African lineage infecting our study population causes both fatal and survival cases. Additionally, CHIKV infection impairs the integrity of the blood-brain barrier, as evidenced by an increase in permeability and altered tight junction protein expression. Overall, our findings improve the understanding of CHIK pathophysiology and the causes of fatal infections.

## Highlights

• Host and viral factors that are associated with chikungunya fatalities are defined
• Chikungunya deaths involve the infection of multiple organs and severe inflammation
• Chikungunya virus crosses the blood-brain barrier
• Chikungunya deaths are associated with dysregulated hemostasis and immune response

## Graphical abstract

**Figure:**
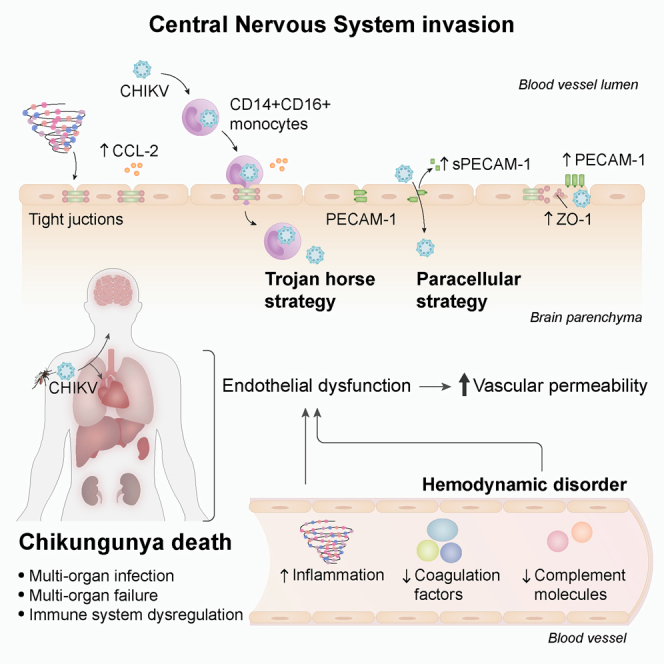

## Teaser

de Souza et al. show the complex factors behind fatal chikungunya cases, highlighting multi-organ infection, central nervous system damage, and severe inflammation. The findings suggest the chikungunya virus crosses the blood-brain barrier, contributing to central nervous system infection. This comprehensive study sheds light on chikungunya pathophysiology and fatal outcomes.

## Introduction

Chikungunya virus (CHIKV) is a mosquito-borne alphavirus that has caused over 10 million cases in more than 125 countries or territories over the last two decades.^1^ Currently, 1.3 billion people live in tropical and subtropical areas at risk for CHIKV transmission.^2^ Recent outbreaks in the southern cone of South America (e.g., Argentina, Paraguay, and Uruguay)^3^^,^^4^ support predictions that climate change is likely expanding the CHIKV distribution, putting more highly populated temperate regions, including the United States of America, China, and continental Europe, at increased risk for epidemic transmission.^5^ In November 2023, the first vaccine against chikungunya (CHIK) was approved by the U.S. Food and Drug Administration.

CHIK is characterized by acute, subacute, or chronic disease. Most cases present with acute fever, myalgia, headache, rash, and arthralgia lasting up to 3 weeks.^6^^,^^7^ Subacute cases are usually characterized by fatigue and articular symptoms between 21 and 90 days after symptom onset. In some CHIK cases, arthralgia can persist for months or years with debilitating polyarthralgia and polyarthritis.^8^^,^^9^ Other complications, such as ophthalmologic, cardiac, and neurological involvement, have also been described in CHIK patients.^1^^,^^7^^,^^10^^,^^11^

Since 2004, the spread of CHIKV has been associated with explosive outbreaks in India and other parts of Asia, the Indian Ocean basin, southern Europe, and, more recently, in Latin America.^12^^,^^13^ The case-fatality ratio (CFR) for CHIK in La Reunion, Southeast Asia, and Brazil has been estimated between 1 and 1.3 deaths per 1,000 cases, similar to that observed during dengue epidemics.^4^^,^^14^ CHIK deaths were associated with sepsis and irreversible multiple organ dysfunction syndrome and more frequent in neonates and elderly people in Brazil, Puerto Rico, and Guadeloupe.^12^^,^^15^^,^^16^

To date, several studies have addressed CHIKV-host interactions and CHIK pathogenesis and pathophysiology.^17^^,^^18^^,^^19^^,^^20^^,^^21^ However, the characteristics and mechanisms associated with fatal outcomes remain poorly understood, partly because most fatal cases have been described only briefly in case reports or systematic reviews.^22^^,^^23^^,^^24^ Here, we combine epidemiological, virological, histopathological, cytokine response, molecular dynamics, metabolomics, proteomics, and genomic analyses to investigate the viral and host factors associated with CHIK deaths.

## Results

### Spatiotemporal dynamics of CHIK deaths in Brazil

**Figure 1:**
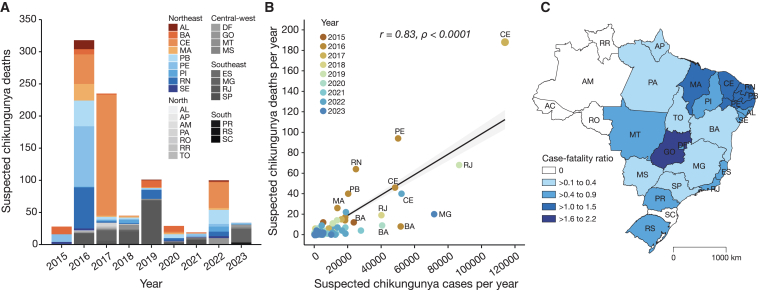
Epidemiology of chikungunya deaths in Brazil (A) Number of suspected chikungunya deaths per year from January 2015 to June 2023. (B) Pearson’s correlation between suspected chikungunya deaths per year and suspected chikungunya cases per year from January 2015 to June 2023, with key states labeled. (C) Map colored according to the cumulative case-fatality ratio of chikungunya deaths per state from January 2015 to June 2023. AC, Acre; AL, Alagoas; AM, Amazonas; AP, Amapá; BA, Bahia; CE, Ceará; ES, Espírito Santo; DF, Distrito Federal (Federal District); GO, Goiás; MA, Maranhão; MG, Minas Gerais; MS, Mato Grosso do Sul; MT, Mato Grosso; PA, Pará; PB, Paraíba; PE, Pernambuco; PI, Piauí; PR, Paraná; RJ, Rio de Janeiro; RN, Rio Grande do Norte; RO, Rondônia; RR, Roraima; RS, Rio Grande do Sul; SC, Santa Catarina; SE, Sergipe; SP, São Paulo; TO, Tocantins; CHIK, chikungunya; km, kilometers.

To estimate the burden of CHIK deaths in Brazil, we analyzed suspected fatal CHIK cases reported between January 2015 and June 2023. A total of 909 suspected CHIK deaths were reported to the Brazilian Ministry of Health across 22 of 27 states (Figure 1A). We found a positive correlation between the total number of suspected CHIK cases and the number of suspected CHIK deaths per year during the annual epidemics in Brazil (Pearson’s r = 0.83, p < 0.0001; Figure 1B). The national cumulative CFR was 0.8 deaths per 1,000 cases, ranging from 0 to 2.2 deaths per 1,000 cases among Brazilian states (Figure 1C). Northeastern Brazil accounted for 63.9% (749,783 of 1,173,659) of suspected CHIK cases reported, and 76.1% (692 of 909) of suspected CHIK deaths reported in Brazil between 2015 and 2023 (Figure 1A). Ceará State had 30.6% (278 of 909) of all suspected CHIK deaths reported from 2015 to 2023, mainly from the 2017 CHIKV epidemic, accounting for 188 suspected CHIK deaths (Figures 1A and 1B). The cumulative CFR in Ceará State from 2015 to 2023 was 1.3 deaths per 1,000 cases (Figure 1C).

### CHIK deaths are associated with multi-organ infection, central nervous system damage, and hemodynamic disorder

**Figure 2:**
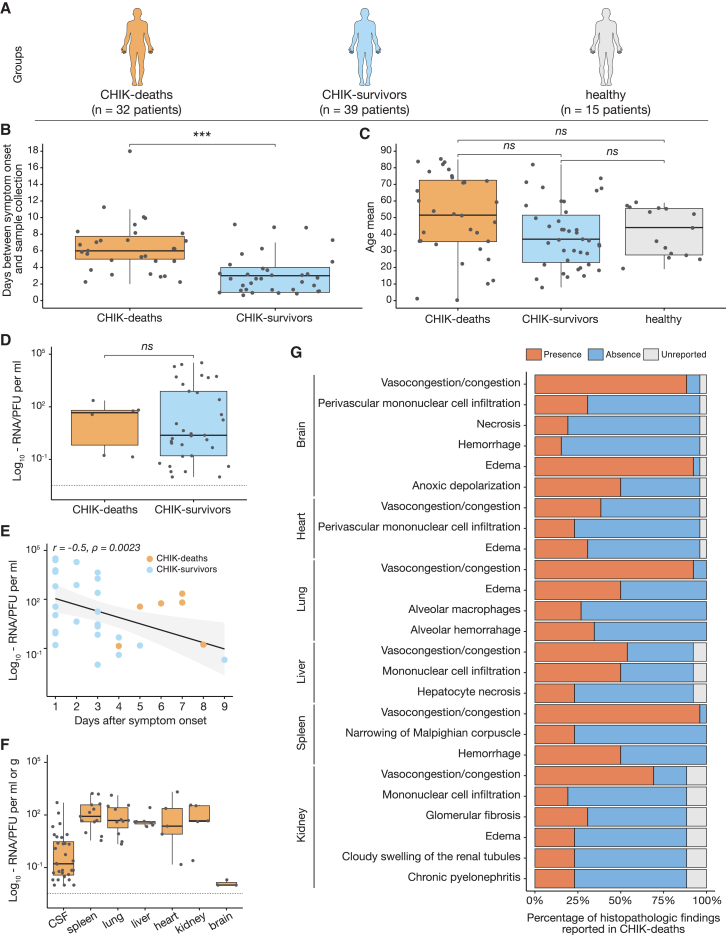
Study design, viral load, and histopathological analyses (A) Study design representing the patient groups evaluated. (B) Days between symptom onset and sample collection. The p values were calculated using the Welch’s t test (30 CHIK deaths versus 35 CHIK survivors). (C) Age distribution of chikungunya patients and controls is shown in years. The p values were calculated using the Wilcoxon signed-rank test with the Dunn-Bonferroni post hoc test. (D) CHIKV RNA concentration (PFU equivalents/mL) in serum as determined by RT-qPCR in CHIK survivors (n = 36) and CHIK deaths (n = 6). (E) Correlation between viremia and time from symptom onset to sample collection samples in days. The correlation was calculated using Spearman’s rank correlation coefficient. (F) CHIKV RNA load (PFU equivalents/grams of tissue) as determined by RT-qPCR in tissue samples of CHIK deaths: CSF (n = 32), spleen (n = 13), lung (n = 11), liver (n = 7), heart (n = 5), kidney (n = 5), and brain (n = 3). (G) Histopathologic findings from the necropsy samples of CHIK deaths (n = 26). Horizontal axes show the percentage of histopathologic findings reported in each fatal chikungunya case. The bar in the boxplots represents the median (middle line), the upper and lower limits represent the 75^th^ and 25^th^ percentiles, and minimum and maximum values (whiskers). Dots represent individual patients. Statistical significance is ^∗∗∗^p < 0.001, ^∗∗^p < 0.01, and ^∗^p < 0.05; ns, not significant. CHIK, chikungunya; PFUs, plaque-forming units; CSF, cerebrospinal fluid; RT-qPCR, reverse transcription quantitative polymerase chain reaction.

To investigate the viral and host factors associated with CHIK deaths, we performed real-time reverse transcription quantitative polymerase chain reactions (RT-qPCR), histopathology, cytokine quantification, metabolomic, proteomic, and viral genomic analyses of serum, cerebrospinal fluid (CSF), and/or tissue samples from 32 individuals who died from acute CHIKV infection (CHIK deaths). These samples were collected at the time of death from a subset of the largest cohort of fatal cases described in 2017 in Ceará State, Brazil.^12^ Additionally, we conducted analyses on serum samples from 39 patients with self-limiting CHIK in Brazil; these patients survived, and none developed chronic disease (CHIK survivors). For comparison, we also included serum samples from 15 blood donors (healthy) who lacked symptoms of viral infection and were negative for CHIKV infection by RT-qPCR and serological tests (Figure 2A). All 86 samples also tested negative for Zika virus (ZIKV) and dengue virus RNA (DENV) by RT-qPCR.^25^^,^^26^

Fever and myalgia were the most frequent signs and symptoms reported in patients from the CHIK-deaths group (78.1% and 59.4%, respectively). In comparison, fever and arthralgia were the most frequently reported in the CHIK-survivors group (69.2% for both). In addition, 21.9% (7 of 32) of patients from the CHIK-deaths group developed neurologic signs and symptoms (e.g., confusion, syncope, adynamia, and sensory loss) (Figures S1A and S1B). The median interval between symptom onset and sample collection was 6 days (interquartile = 5 and 7.8 days) in the CHIK-deaths group and 3 days (interquartile = 1 and 4 days) in the CHIK-survivors group (Figure 2B). The CHIK-deaths group was composed predominantly of males (male-to-female ratio = 1.46), and the median age was 52 years (interquartile, 36–73 years old), but no significant difference regarding age and sex were observed among the three groups (Figures 2C and S1C). The comorbidity most often reported in the CHIKV-deaths group was hypertension (21.9%, 7 of 32) (Figure S1D).

There were no statistically significant differences in viremia levels between the CHIK-death and -survivor groups (Figure 2D). Viremia was highest early in the course of the disease and decreased over time (Figure 2E). CHIKV RNA was detected in CSF samples from all fatal cases (Figure S1E). CHIKV RNA was also detected in the spleen (52%, 13 of 25), lung (44%, 11 of 25), liver (28%, 7 of 25), kidney (20%, 5 of 25), heart (20%, 5 of 25), and brain (13%, 3 of 23) of CHIK-death patients from available specimens (Figures 2F and S1E). Moreover, histopathological findings of CHIK-death cases revealed extensive damage in the brain (e.g., anoxic depolarization, perivascular mononuclear infiltration, necrosis, and hemorrhage), and hemodynamic abnormalities (e.g., vascular congestion and edema) in the brain, lung, spleen, and kidney (Figures 2G and S2).

### Cytokine and metabolome profiles suggest exacerbated pro-inflammatory responses and hemodynamic shock in CHIK deaths

**Figure 3:**
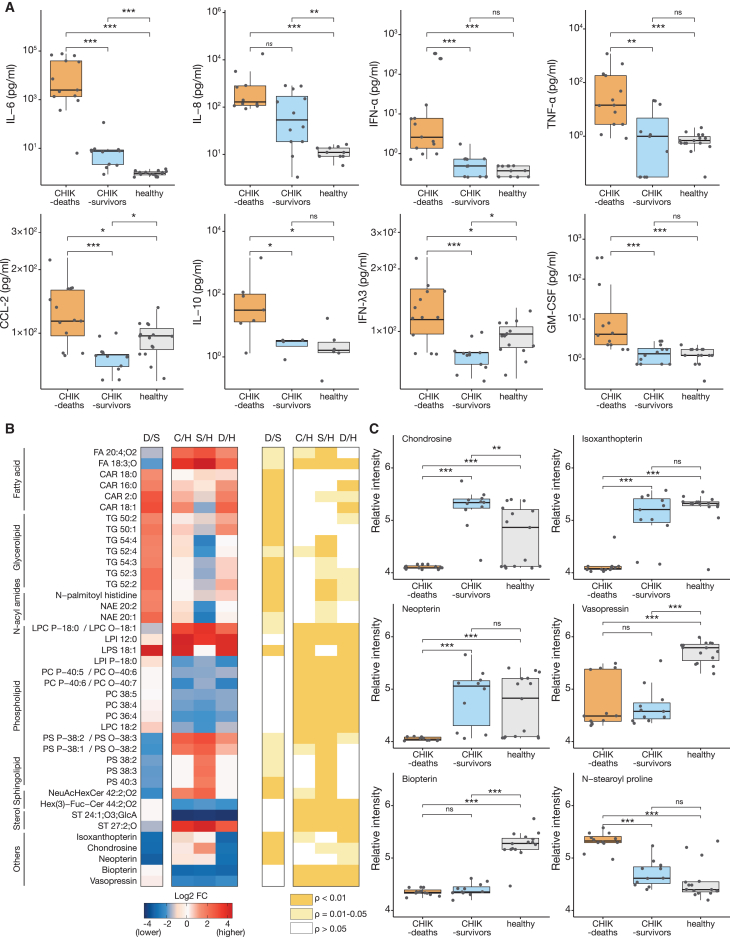
Cytokine responses and metabolomic signatures of chikungunya patients (A) Serum cytokine profiles of chikungunya patients (fatalities, n = 13, and survivors, n = 12) were compared with healthy controls (n = 15). Statistical analyses were performed using the Wilcoxon signed-rank test with the Dunn-Bonferroni post hoc test. IFN, interferon; IL, interleukin; TNF, tumor necrosis factor; GM-CSF, granulocyte-macrophage colony-stimulating factor; CCL-2, chemokine ligand 2. (B) Heatmap analysis of selected metabolite markers for chikungunya patients (D, deaths; S, survivors; C, chikungunya cases from both outcomes) compared with healthy blood donors (H) shows the log_2_ FC and the p value. The log_2_-fold changes (log_2_ FCs) are scaled from blue (low intensity) to red (high intensity). Statistical analyses were performed using the Wilcoxon test in MetaboAnalyst.^27^ (C) The expression level change (normalized log_10_-transformed ion intensity) of eight selected regulated metabolites with significant differences between chikungunya patients and healthy controls. Statistical analyses were performed using the Wilcoxon sign-rank test with the Dunn-Bonferroni post hoc test. The bar in the boxplots represents the median (middle line), the upper and lower limits represent the 75^th^ and 25^th^ percentiles, and minimum and maximum values (whiskers). Dots represent individual patients. Statistical significance is ^∗∗∗^p < 0.001, ^∗∗^p < 0.01, and ^∗^p < 0.05; ns, not significant.

To evaluate the immune system response in fatal and survivor CHIK cases and compare them to the healthy blood donor group, we quantified the systemic levels of cytokines and chemokines. We found that the sera of the CHIK-deaths group had significantly higher levels of pro-inflammatory cytokines and chemokines than those of CHIK survivors and/or healthy persons, including interleukin (IL)-6, IL-8, interferon (IFN)-α, tumor necrosis factor alpha (TNF-ɑ), granulocyte (GM)-CSF, and C-C motif chemokine ligand 2 (CCL-2) (Figure 3A). Also, the mean anti-inflammatory IL-10 in the CHIK-deaths group was 9.7- and 17.8-fold higher than CHIK-survivor and healthy groups (p = 0.042 and p = 0.045, respectively). Moreover, IFN-λ3 (IL-28B) was 1.4-fold higher in the CHIK-deaths group compared with the CHIK-survivors group (p = 0.0005) (Figure 3A; Table S1).

We next used a shotgun-metabolomic approach to characterize the serum profile of CHIK patients with different disease outcomes, and compared patterns to those from healthy blood donors. Based on an analysis of 82 metabolites, we found 56 that were differentially expressed in the sera of the CHIK-deaths group compared with patients who survived CHIK (Figures S3 and S4). Most of these differentially expressed metabolites were associated with membrane formation, intracellular signaling, and hemodynamic and homeostasis processes. For example, we found higher levels of several fatty acids (carnitines), glycerolipids (TG), and N-acyl amides in the CHIK deaths compared with the CHIK-survivors group. By contrast, we observed lower levels of phosphatidylserine (PS) in the CHIK deaths compared with CHIK-survivors group, but a higher level in the CHIK survivors compared with the healthy group. In addition, we found that phosphatidylcholine (PC) levels were lower in both CHIK outcomes (fatal and survivors) compared with the healthy group (Figure 3B). In particular, we identified a decrease of chondrosine (1.2-fold), isoxanthopterin (1.3-fold), and neopterin (1.2-fold) in CHIK deaths compared with CHIK-survivors and healthy groups (Figure 3C). We also observed a significant reduction of vasopressin and biopterin levels in CHIK cases from both outcomes compared with the healthy control group. In addition, we found that N-stearoyl proline levels were 1.2-fold higher in the CHIK-deaths group compared with CHIK-survivors and healthy groups (Figure 3C). Overall, these findings suggest that metabolic dysregulation may contribute to the development of severe CHIKV infection and death.

### Proteomic profiles suggest dysregulation in hemostasis and immune system pathways in CHIK deaths

**Figure 4:**
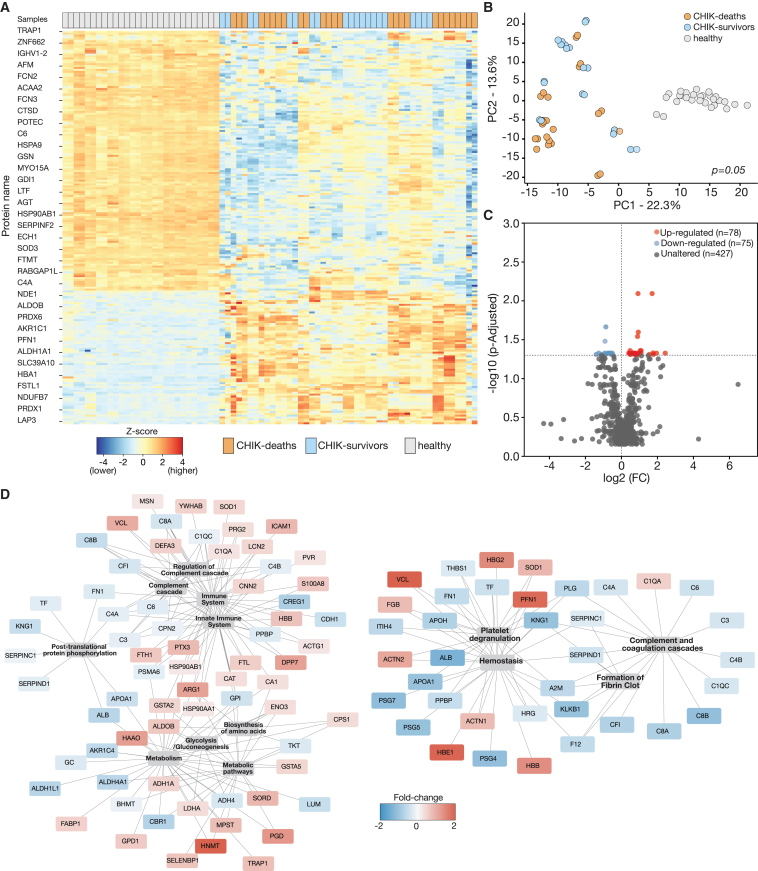
Proteomic signatures of chikungunya patients with different disease outcomes (A) The proteomic profiles of the most significantly differentially expressed proteins in chikungunya patients (deaths, n = 13, and survivors, n = 11) and healthy controls (n = 15). The color intensity is related to the normalized abundance for each protein. All proteomic analyses were performed in technical duplicate. The relative intensities (*Z* score) for each are scaled from blue (low intensity) to red (high intensity). Samples were clustered according to Pearson correlation coefficient distance. (B) Principal-component analysis of the proteomic data from the serum samples of chikungunya patients (i.e., deaths and survivors) compared with blood donors (healthy). Each dot represents one patient and the respective technical replicate, color-coded for the different groups described in the figure. (C) Volcano plot displaying the log_2_-fold change (x axis) against the t test-derived −log_10_ statistical p value (y axis) for all proteins differentially expressed between chikungunya deaths and chikungunya survivors. Proteins with significantly decreased levels (p < 0.05) are shown in blue, and proteins with significantly increased levels are noted in red. The gray circles indicate unaltered proteins. All the proteins are shown. (D) Protein-protein interaction analysis using proteins related to the hemostasis pathway (hemostasis, platelet degranulation, complement and coagulation, and formation of fibrin clot) and immune system pathways (neutrophil degranulation, biosynthesis of amino acids, glycolysis/gluconeogenesis, post-translational protein phosphorylation) from chikungunya deaths compared with chikungunya survivors. Gene symbols (abbreviation names) for protein-coding genes followed the nomenclature approved by the HUGO Gene Nomenclature Committee.^28^

To characterize the proteomic profiles in CHIK cases, we performed mass spectrometry (MS)-based shotgun proteomics (liquid chromatography [LC]-MS) on serum from fatal and survivor outcomes and compared them independently with the healthy control group. Hierarchical cluster analysis of the protein abundances revealed significant and distinct proteomic profiles between the CHIK patients for both fatal and survivor outcomes compared with the healthy group, suggesting a widespread remodeling of proteome regulation in response to CHIKV infection (Figure 4A). The principal-component analysis (PCA) showed a notable overlap in proteomic signature between CHIK-deaths and CHIK-survivor groups compared with healthy blood donors (Figure 4B). We identified and quantified 580 proteins (Figure S5), including 338 proteins (188 downregulated and 150 upregulated) that were differentially expressed in the CHIK-deaths group compared with the healthy group. Also, we found 525 proteins (268 downregulated and 257 upregulated) differentially expressed in the CHIK-survivors group compared with the healthy group (Figure S5). In addition, we found 153 proteins (75 downregulated and 78 upregulated) that differentiated CHIK deaths from CHIK-survivor groups (Figure 4C).

To better understand the impact of CHIKV on specific biological processes, we conducted a pathway enrichment analysis using significantly dysregulated proteins and the Kyoto Encyclopedia of Genes and Genomes (KEGG) and Reactome databases.^29^^,^^30^ We found that enriched pathways associated with CHIK-deaths and CHIK-survivor groups but not with the healthy group were related to hemostasis (i.e., platelet degranulation, complement and coagulation cascades, common pathway of fibrin clot formation, clotting cascade, platelet activation and aggregation, binding and uptake of ligands by scavenger receptors, and metabolism), and the immune system (innate immune system, neutrophil degranulation, biosynthesis of amino acids, glycolysis/gluconeogenesis, and post-translational protein phosphorylation) (Figures S6 and S7). We also found 36 dysregulated proteins (26 downregulated and 10 upregulated) that are involved in hemostasis (platelet degranulation, complement and coagulation cascades, clotting cascade) and immune pathways (neutrophil degranulation, biosynthesis of amino acids, glycolysis/gluconeogenesis, post-translational protein phosphorylation) that were altered in CHIK deaths compared with CHIK-survivors groups (Figure 4D). For example, proteins critical to inflammatory processes, such as intercellular adhesion molecule 1 (ICAM-1), calponin 2 (CNN2), S100 calcium-binding protein A8 (S100A8), and dysregulation of proteins involved with neutrophil degranulation (pentraxin 3 [PTX3], pro-platelet basic protein [PPBP], cellular repressor of E1A stimulated genes 1 [CREG1], proteoglycan 2 pro eosinophil major basic protein [PRG2], and dipeptidyl peptidase 7 [DPP7]) were upregulated in CHIK deaths compared CHIKV-survivors. We also observed dysregulation of many proteins involved in fibrin clot formation (i.e., coagulation cascade), including downregulation of coagulation factor XII (F12), kallikrein B1 (KLKB1), kininogen 1 (KNG1), plasminogen (PLG), alpha-2-macroglobulin (A2M), SERPIN family members (SERPINC1 and SERPIND1), fibrinogen beta chain (FGB), and complement system proteins (C3, C4A, C4B, C1QC, C6, C8A, and C8B). In the platelet degranulation (hemostasis) pathway, we detected upregulation of actin proteins (vinculin [VCL], profilin 1 [PFN1], actin gamma 1 [ACTG1], actinin alpha 1 and 2 [ACTN1 and ACTN2]), and hemoglobin subunits (HBB, HBE1, and HBG2). Collectively, these results indicate that many specific affected pathways are similar in both fatal and survivor outcomes, but there is differential dysregulation of hemostasis and immune system activation between the CHIK-deaths and CHIK-survivor groups.

### CHIK cases were caused by the East-Central-South-African lineage, and a single mutation in the E2 envelope glycoprotein may increase virion binding to MXRA8 receptor

**Figure 5:**
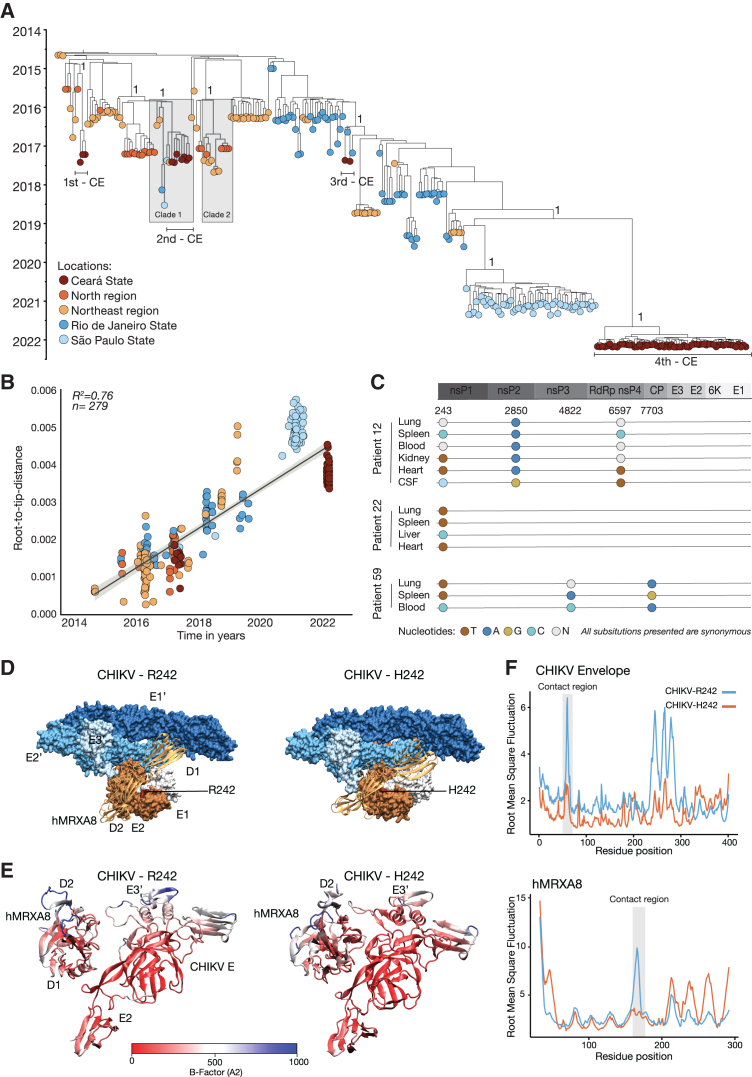
Phylogenetic, intrahost diversity, and molecular dynamics of CHIKV-ECSA in Brazil (A) Time-rooted phylogenetic tree of the CHIKV East-Central-South African lineage in Brazil (n = 279), including 45 new sequences from this study. Tips are colored according to the source region or state of each sample. (B) Regression of sequence sampling dates against a root-to-tip genetic distance in a maximum likelihood phylogeny of the CHIKV-ECSA lineage in Brazil. Sequences are colored according to five locations (Ceará, Rio de Janeiro, and São Paulo States, and North and Northeast regions of Brazil). (C) Intrahost single nucleotide polymorphisms associated with three chikungunya deaths (patients 12, 22, and 59). (D) The E2-R242H substitution is shown in the complex structure of the CHIKV E3-E2-E1 glycoprotein spike bound to the human MXRA8 receptor. The 90° rotation on the right panel shows the top view of the complex. (E) Average structures for the E2-E3-hMRXA8 complex that appear in the ancestral and mutant strains, colored according to the beta temperature factor of each residue. Red regions correspond to low-mobility regions, whereas blue regions correspond to high-mobility regions. (F) Root mean square fluctuation plots for the ancestral E2-R242 (blue line) and the E2-H242 substitution (red line) systems regarding their E2-E3 proteins and the hMRXA8 receptor. Residues located in the contact region are highlighted for both structures. Non-structural (ns) proteins. C-capsid; E-envelope. RdRp, RNA-dependent RNA polymerase; T, thymine; A, adenine; C, cytosine; G, guanine; N, undetermined based.

To assess how viral factors might impact the clinical CHIK outcome, we used next-generation sequencing to generate 46 novel CHIKV genomes from serum samples of CHIK-survivors (n = 23 individuals) and CHIK-deaths groups (n = 12 individuals). To contextualize our data, we also included 10 novel CHIKV genome sequences obtained from patients in São Paulo, Maranhão, and Alagoas States in Brazil (Table S2). All CHIKV genomes generated had >75% coverage with a mean depth of at least 20-fold per nucleotide (Figure 5A). We confirmed that our genomic dataset had a strong temporal signal based on regression of genetic divergence from root-to-tip against sample collection dates (Figure 5B). Our phylogenetic analysis confirmed that the East-Central-South African (ECSA) lineage, one of two major lineages circulating in the Americas (along with the Asian lineage), caused CHIK cases, resulting in both fatal and survivor outcomes. Next, we found that CHIKV sequences associated with the 2017 outbreak in Ceará State resulted from at least three independent introductions in late 2016 (Figure 5A). The earliest introduction in Ceará State was estimated to have occurred around mid-August 2016 (clade CE1, 95% Bayesian credible interval = 19 October 2015 to 23 November 2016) and included three genomes from fatal CHIKV cases. The second introduction around early October 2016 (clade CE2, 95% Bayesian credible interval = 27 March 2016 to 5 February 2017) had six fatal CHIK cases. The third introduction was represented with two CHIKV genomes from fatal CHIK cases that likely occurred in late 2015 and late 2016 (Figure 5A). Additionally, to identify potential single nucleotide polymorphisms (SNPs) associated with tissue tropism in the fatal CHIKV cases, we sequenced CHIKV from multiple tissues and biological fluids of three CHIK deaths, and we identified up to three SNPs in multiple samples from the same patient, all of which were synonymous (Figure 5C).

We probed potential genomic mutations associated with fatal CHIK patients, and we identified one amino acid change from arginine (R) to histidine (H) at residue 242 of the E2 envelope glycoprotein (E2-H242) (Protein Data Bank, PDB: 6JO8, and nucleotide position 9,079 using GenBank KY055011 as reference) in the two clades, clade 1 and clade 2. Clade 1 included sequences from 7 CHIK deaths in Ceará State and 5 sequences from other locations, whereas clade 2 included 11 sequences from CHIK survivors in Pará, Amazonas, and Maranhão States (Figure 5A), demonstrating that E2-H242 was present in both CHIKV fatal and survivor outcomes. Our analysis showed that the E2-H242 substitution emerged convergently in clade 1 and clade 2 between late 2015 and mid-2016 (Figure 5A), suggesting that it mediated increased viral fitness. Because of this, we conducted molecular dynamic simulations of CHIKV with the matrix remodeling associated 8 (MXRA8), a receptor for multiple arthritogenic alphaviruses, including CHIKV.^31^^,^^32^^,^^33^ We observed that the CHIKV-ECSA lineage with H242 resulted in reduced E2 surface area and increased the buried surface area interacting with the human MXRA8 receptor, compared with the ancestral E2-R242, due to physical proximity of the E3 protein toward the D2 domain of the MXRA8 receptor (Figures 5D, 5E, and S8). Also, the CHIKV-H242 variant resulted in lower root mean square fluctuation compared with CHIKV-R242 in the contact region of E2 protein with MXRA8 (Figures 5F and S8). Lastly, the resulting binding free-energy values obtained for the CHIKV-R242 were −375 ± 1 kcal/mol, compared with −395 ± 2 kcal/mol for CHIKV-H242, suggesting an increase in receptor binding affinity. Alanine-scanning mutagenesis analysis also indicates that E2-H242 may be relevant to the MXRA8 interaction with CHIKV glycoprotein based on free-energy differences compared with CHIKV strains harboring R242 (Figure S9). Collectively, the E2-R242H substitution data suggested its association with increased viral fitness linked with the large 2017 epidemic in Ceará State; however, this mutation did not appear to be associated with an increased risk of death since it was identified in patients with both fatal and survival outcomes.

### CHIKV crosses the BBB into the brain parenchyma

To investigate the mechanisms by which CHIKV breaches the blood-brain barrier (BBB) and gains access to the brain parenchyma, as indicated by the autopsy samples, we employed an *in vitro* model of the human BBB and examined the effects of CHIKV infection on peripheral blood mononuclear cell (PBMC) transmigration and BBB integrity. We first examined the role of CCL-2, a critical chemokine involved in BBB transient disruption and leukocyte transmigration into the brain,^34^ as histopathological analysis of fatal CHIKV cases revealed perivascular mononuclear cell infiltration in the brain (Figure 2G). Consistent with this observation, we found that CHIKV-infected individuals who succumbed to the disease had significantly higher serum levels of CCL-2 compared with both survivors and healthy controls (Figure 3A).

**Figure 6:**
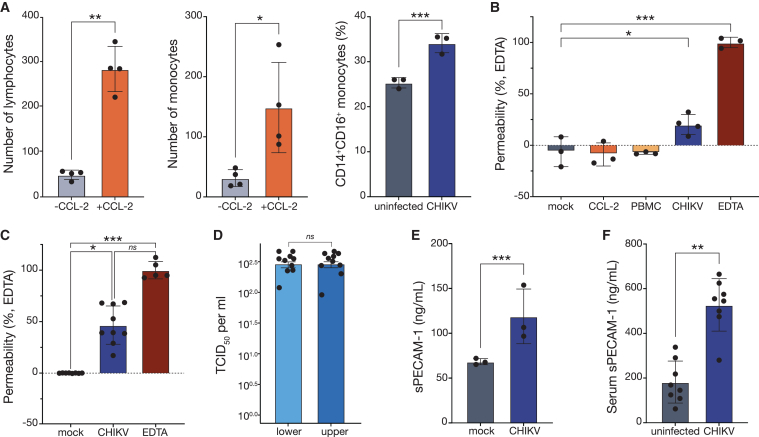
Leukocyte transendothelial migration during chikungunya virus infection in a human blood-brain barrier model (A) Quantification of the number of lymphocytes (CD45^+^), monocytes (CD14^+^), and CD14^+^CD16^+^ monocytes migrating from the luminal (upper) to the abluminal (lower) chamber during 24 h. PBMCs infected with CHIKV at a multiplicity of infection (MOI) of 0.1, in the presence or absence of CCL-2 at 100 ng/mL. Fluorescence-activated cell sorting was used to identify lymphocytes (CD45^+^), monocytes (CD14^+^), and CD14^+^CD16^+^ monocytes, and CHIKV-infected cells. (B) Percentage of permeability of the BBB model, assessed by quantifying the passage of albumin conjugated to Evans blue dye through cocultures of primary human brain microvascular endothelial cells (BMVECs) and human primary astrocytes. Mock is only cocultures. CCL-2 is cocultures plus CCL-2 at 100 ng/mL. PBMC is peripheral blood mononuclear cells (PBMCs), cocultures, and CCL-2 at 100 ng/mL. CHIKV is PBMC infected with CHIKV at MOI of 0.1 plus cocultures, and CCL-2 at 100 ng/mL. EDTA is coculture and treatment with 4 mM of ethylenediaminetetraacetic acid (EDTA) as a positive control for disruption of the BBB. The data are expressed as a percentage of permeability related to EDTA. (C) Percentage of permeability of the BBB model, assessed by quantifying the passage of fluorescein isothiocyanate (FITC)-dextran 4kDa through cocultures of BMVECs and human primary astrocytes. Uninfected is only coculture. CHIKV is coculture infected with CHIKV at MOI of 0.1 PFU/cell. EDTA is coculture and treatment with 4 mM of EDTA. (D) Viral titration was measured in the luminal and abluminal chambers of the trans-well plates by TCID_50_ assay. (E) Levels of soluble platelet endothelial cell adhesion molecule 1 (sPECAM-1) in media from PBMCs infected with CHIKV compared with uninfected PBMCs. (F) Serum levels of sPECAM-1 in uninfected individuals (n = 8) and CHIK patients (n = 8). Data are presented as the mean ± standard deviation. Statistical analyses were performed using the paired t test, one-way ANOVA, and Kruskal-Wallis test. Statistical significance is ^∗∗∗^p < 0.001, ^∗∗^p < 0.01, and ^∗^p < 0.05; ns, not significant. Dots represent individual biological replicates. −CCL-2, absence of CCL-2. +CCL-2, presence of 100 ng/mL of CCL-2.

Based on our *in vitro* BBB model, we investigated the transmigration of CHIKV-infected PBMCs in response to CCL-2 by adding CHIKV-infected PBMCs to cocultures supplemented with CCL-2. We demonstrated that uninfected or CHIKV-infected lymphocytes or monocytes did not transmigrate in the absence of CCL-2 (Figure 6A). CCL-2 significantly increased the transmigration of CHIKV-infected lymphocytes and monocytes across the BBB (p = 0.0040 and 0.0379, respectively, Figure 6A). Notably, CCL-2 mediated transmigration of monocytes exposed to CHIKV, specifically the CD14^+^CD16^+^ monocytes (p = 0.0001), a more mature subpopulation of pro-inflammatory monocytes, to cross the BBB at a significantly higher than uninfected cells (Figure 6A). Subsequently, we assessed the impact of CHIKV-infected leukocyte transmigration on BBB permeability. Although mock (coculture), CCL-2 alone, and PBMCs alone did not alter permeability (Figure 6B), the combination of CHIKV-infected PBMCs and CCL-2 induced a 20% increase in endothelial permeability (p = 0.0131), suggesting a synergistic effect between CHIKV infection and CCL-2 signaling to increase BBB permeability.

To determine whether CHIKV infection alone could compromise BBB permeability, we performed the transmigration experiment using our *in vitro* BBB model in the absence of CCL-2 or PBMCs. Remarkably, CHIKV infection alone induces a 39% increase in BBB permeability at 24 h post-infection compared with uninfected cells, demonstrating the virus's capacity to independently enhance BBB permeability (p = 0.0212, Figure 6C). This finding was corroborated by the detection of infectious CHIKV in the supernatant of both the upper (luminal) and lower (abluminal) chambers of the *in vitro* BBB model at 24 h post-infection, with mean viral titers of 3.2 × 10^2^ TCID_50_/mL in both chambers, confirming the virus's ability to cross the endothelial barrier (Figure 6D).

Platelet/endothelial cell adhesion molecule-1 (PECAM-1) is a key adhesion molecule involved in transmigration and BBB integrity,^35^ so we investigated it as a potential mechanism underlying CHIKV-mediated BBB disruption. Notably, we observed a 1.5-fold increase in soluble PECAM-1 (sPECAM-1) levels in the media from our *in vitro* transmigration experiments compared with uninfected cells (p = 0.0007, Figure 6E), suggesting a PECAM-1 shedding event similar to that observed with other neurotropic viruses such as human immunodeficiency virus (HIV).^36^ Similarly, serum levels of sPECAM-1 were significantly elevated (3.5-fold) in CHIKV-infected individuals compared with uninfected controls (p = 0.0061, Figure 6F), indicating that sPECAM-1 shedding may play a role in CHIKV-associated BBB dysfunction *in vivo*. Overall, our findings demonstrate that CHIKV-infected leukocytes or CHIKV alone can cross the BBB in a manner dependent on CCL-2 and PECAM-1 in our *in vitro* BBB model, which provides insights into the mechanisms by which CHIKV gains access to the CNS and potentially contributes to neurological complications.

### CHIKV disrupts the integrity of the BBB

**Figure 7:**
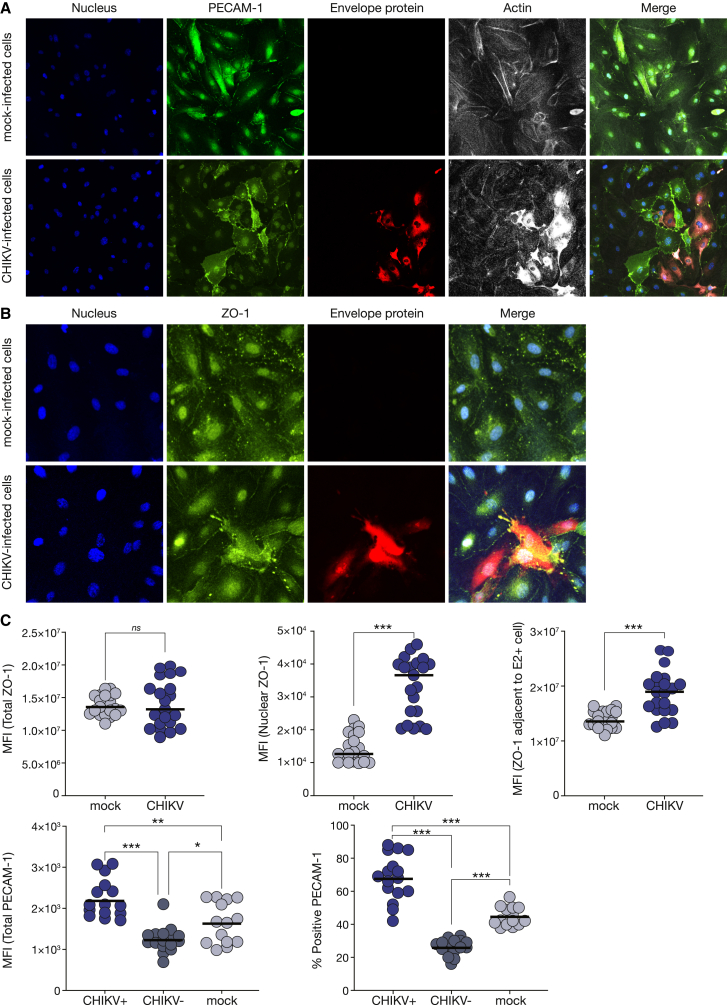
Disruption of primary human brain microvascular endothelial monolayer during chikungunya virus infection (A) Immunofluorescence analysis of PECAM-1 in CHIKV-infected (MOI 0.1, 24 h) primary human brain microvascular endothelial cells (BMVECs) at 90% confluence. Co-staining with 4,6-diamidino-2-phenylindole (DAPI) labels the nucleus (blue), CHIKV-infected infected cells (red), and actin (gray). Areas of colocalization (merge) appear yellow. (B) Immunofluorescence analysis of ZO-1 (green) in CHIKV-infected (MOI 0.1, 24 h) BMVECs at 90% confluence. Co-staining with DAPI labels the nucleus (blue), and CHIKV-infected infected cells labeled CHIKV-envelope protein antibody (red). Areas of colocalization (merge) appear yellow. (C) Quantitation of ZO-1 and PECAM-1 mean fluorescence intensity (MFI) of ten individual representative regions of interest (ROIs). Total ZO-1 expression (upper left), nuclear ZO-1 expression (upper middle), ZO-1 expression in membrane cells adjacent to E2-CHIKV-positive cells (upper right), total PECAM-1 (bottom left), and percentage of positive PECAM-1 (bottom right) in CHIKV-positive cells (E2-positive), CHIKV-negative cells (E2-negative), and uninfected cells. Data are presented as the mean ± standard deviation. Statistical analyses were performed using the paired t test and one-way ANOVA. Statistical significance is ^∗∗∗^p < 0.001, ^∗∗^p < 0.01, and ^∗^p < 0.05; ns, not significant.

To further investigate the impact of CHIKV infection on endothelial monolayer integrity, we conducted immunofluorescence analyses of primary human brain microvascular endothelial cells (BMVECs) under uninfected (mock) and CHIKV-infected conditions, specifically examining the expression and localization of PECAM-1 and zonula occludens 1 (ZO-1). ZO-1 is a tight junction-associated protein and also a transcription factor in conditions of BBB disruption.^37^ Our results revealed that a small subset of BMVECs was susceptible to CHIKV infection (Figures 7A and 7B). Moreover, CHIKV infection disrupted the organization of cell junction proteins PECAM-1 and ZO-1 (Figures 7A and 7B). We observed an increase in the nuclear levels of ZO-1, as well as its accumulation in the membranes of cells adjacent to E2-positive CHIKV-infected cells (Figure 7B). Notably, we demonstrated the significant relocalization of ZO-1 during CHIKV infection (Figure 7C). Additionally, endothelial cells exhibited increased expression of PECAM-1 in CHIKV-infected cells, but not in uninfected cells (Figure 7C), further supporting a CHIKV-induced BBB disruption and transmigration phenotype.

## Discussion

In this study, we assessed fatal CHIK by examining both viral and host factors. We also contextualized the CHIK deaths across Brazil by providing a spatiotemporal analysis from 2015 to 2023. Our analysis showed that the cumulative CFR in Brazil was 0.8 deaths per 1,000 cases, which is similar to previous estimates from a high-sensitivity surveillance.^38^ Then, our investigation focused on CHIK cases from Ceará, the Brazilian state with the highest number of cases and deaths. We found that CHIK deaths generally involved multiple organs, CNS infection, and hemodynamic disorders. Our study confirmed that CHIKV can invade the CNS by demonstrating the presence of viral RNA in CSF and brains from fatal cases. This is consistent with previous studies, which have detected CHIKV RNA and isolated the virus from CSF samples, as well as detected CHIKV antigen in the brains of fatal cases.^15^^,^^39^ However, further studies are needed to determine the frequency of CNS infection among patients infected with CHIKV.

Our findings revealed the presence of mononuclear cell infiltrations in the brain, heart, liver, and kidney in CHIK deaths. These infiltrations are likely a consequence of an inflammatory reaction that triggers the migration of lymphocytes and monocytes through the endothelium into these organs. Additionally, our data demonstrated a substantial overproduction of pro-inflammatory cytokines combined with the higher secretion of IL-10 (anti-inflammatory) in the serum of the CHIK-deaths group compared with CHIK-survivor patients. Elevated levels of cytokine in blood could contribute to uncontrolled inflammation and loss of homeostasis of the immune response.^40^ Among inflammatory mediators, we observed significantly higher CCL-2 levels in the serum of the CHIK-deaths group compared with CHIK survivors. Our findings suggest that CCL-2 plays a crucial role in recruiting lymphocytes and monocytes into the brain, including CD14^+^CD16^+^ monocytes that correspond to a more mature population. CD14^+^CD16^+^ monocytes represent a subset of pro-inflammatory monocytes implicated in the pathogenesis of CNS infections due to their ability to induce neuroinflammation and neuronal damage.^36^^,^^41^^,^^42^ Additionally, we demonstrated that CD14^+^CD16^+^ monocytes are susceptible to CHIKV infection and possess the ability to transmigrate across the BBB in our *in vitro* model, suggesting that this monocyte subpopulation could serve as a Trojan horse mechanism for CHIKV entry into the CNS. Similar to previously described mechanisms for HIV and ZIKV infection, CD14^+^CD16^+^ monocytes may contribute to the processes leading to neurological CHIK disease.^41^^,^^42^ Our findings also revealed an increase in sPECAM-1 expression on the leukocyte infected with CHIKV, an increase in expression of PECAM-1 in the membrane of BMVECs infected with CHIKV, and elevated systemic sPECAM-1 shedding during CHIKV infection in patients. These changes may contribute to the paracellular diapedesis of CHIKV-infected leukocytes. We hypothesize that sPECAM-1 production by CHIKV-infected leukocytes leads to the accumulation of sPECAM-1 in the CNS vasculature and the generation of truncated, intracellular forms of PECAM-1 in leukocytes. These alterations can disrupt PECAM-1 homophilic interactions between endothelial cells with endothelial cells and between endothelial cells and leukocytes.^43^ This idea is also supported by the increase of ZO-1 in CHIKV-infected cells, as well as ZO-1 translocation into cellular nuclei. Together, these changes might contribute to the enhanced transmigration of CHIKV-infected leukocytes into the CNS and alterations in BBB permeability during the pathogenesis associated with the CNS.

Immune system dysregulation was also evident in the metabolomic and proteomic profiles of CHIK patients from both outcomes, compared with the healthy group, also with significant differences between fatal and survivor outcomes. Both profiles may help to explain the observed inflammation, endothelial damage, and vascular permeability, corroborating our histopathologic and cytokine response findings. For example, we identified higher expression of ICAM-1, an adhesion molecule that is basally expressed at low levels on endothelial cells, but upregulated in response to inflammatory stimuli.^44^ ICAM-1 has important roles in leukocyte transendothelial migration, regulating leukocyte migration, and interactions with the vessel wall^45^ in a similar manner to PECAM-1. ICAM-1 knockout mice exhibit decreased lethality from West Nile encephalitis due to reduced viral load, leukocyte infiltration, and neuronal damage.^46^ We also found upregulation of several actin proteins essential for regulating cytoskeletal dynamics, such as VCL, CNN2, PFN1, ACTG1, ACTN1, and ACTN2.^47^ In addition, we found a significant reduction in chondrosine, a component of connective tissue that contributes to the organization and resilience of the extracellular matrix,^48^ in sera of CHIK deaths compared with CHIK survivors. The CHIK fatality patients also showed a substantial reduction in isoxanthopterin in the serum, suggesting oxidative stress, as well as liver and renal damage.^49^^,^^50^ We also found a significant increase in N-acyl amides (i.e., anandamide and N-stearoyl-proline) in CHIK patients with fatal outcomes; these metabolites are biologically active, endogenous ligands that bind to transient receptor potential (TRP) channels. Within TRPs, the TRP-vanilloid (TRPV) family exerts a role in mechanisms of pain and inflammation.^51^ We also identified a significant reduction of neopterin in sera of fatal patients compared with survivors and healthy blood donors, suggesting a suppressed cellular immune response in the fatal CHIK patients. Neopterin is synthesized mainly by activated monocytes/macrophages after IFN-γ stimulation released by natural killer cells and T-lymphocytes; thus, the neopterin decrease could be due to the responsiveness of macrophages to IFN-γ, or an increase in the clearance of neopterin from the blood.^52^

Hyperinflammation observed in the CHIKV-deaths group can also lead to endothelial injury, overexpression of tissue factor, and dysregulation of the coagulation cascade. For example, F12 is activated by KLKB1 and accelerated by KNG1 on endothelial cell surfaces^53^; these three proteins were all upregulated in CHIK fatal cases. This is significant because activated F12 then catalyzes F11, which in turn activates F9, leading to thrombosis and fibrin formation.^54^ Notably, three coagulation-associated protease inhibitors (SERPINC1, SERPIND1, and A2M), KNG1, which disrupt the kinin-kallikrein system,^55^ and PLG, which converts and breaks down blood clots,^56^ were also all downregulated in fatal CHIK cases. Additionally, vasopressin levels were reduced in sera of CHIK patients with both outcomes. Vasopressin is an essential metabolite for cardiovascular homeostasis because it constricts blood vessels and increases water reabsorption in the kidney^57^^,^^58^; therefore, it is plausible that CHIKV can cause a hemodynamic disturbance, which in some cases can be more prominent and lead to fatal outcomes. Further research is needed to elucidate the underlying mechanisms of metabolic and proteomic dysregulation in CHIKV infection.

Our analyses indicate at least three CHIKV introductions caused the 2017 epidemic in Ceará State, resulting in fatal outcomes. We also identified certain ECSA CHIKV strains carrying the E2-H242 substitution, which emerged from a convergent evolution process in two independent ECSA lineages in the northern and northeast regions of Brazil. We hypothesize that R242 might be associated with increased binding of the E2 protein with the human MXRA8 receptor^32^ compared with the ancestral residue (E2-R242). However, since this mutation was identified in both fatal and survival cases, it is unlikely to be responsible for fatal outcomes. Indeed, CNS infection and fatal CHIK do not appear to be restricted to a particular CHIKV lineage and have been reported in patients from several regions affected by different CHIKV lineages, such as Asian,^15^^,^^59^ ECSA,^11^^,^^12^^,^^41^ and the Indian Ocean lineage.^60^^,^^61^ Nevertheless, the effects of E2-R242 mutation on CHIKV infection should be further investigated. As expected, our results show a lower viral genetic intrahost diversity of CHIKV in acute infection compared with chronic viral infections (e.g., hepatitis C virus and HIV), similar to that previously described for DENV.^62^

Our study has several limitations. First, our retrospective approach using residual samples could not directly investigate the cellular immune responses in CHIK deaths due to a lack of PMBC collections. Second, our correlation of CHIKV viremia and cytokine profiles in fatal and survivor outcomes was based on matched intervals between symptom onset and sample collection up to 21 days (i.e., acute CHIK phase). Because severe cases could differ from milder outcomes in the timing of the initial presentation for care, further studies should be conducted to investigate this correlation to clarify the potential association between viremia, cytokine response, and acute CHIK outcomes. Third, the metabolites and proteins that might be useful biomarkers for CHIK outcomes must be further validated using a large cohort of patients, including serum samples from chronic CHIK cases and other endemic arbovirus infections (e.g., DENV and ZIKV). Finally, our retrospective study did not attempt to describe the entire spectrum or mechanisms of CHIK deaths but did shed light on unexplored pathophysiology of fatal outcomes for further study. Thus, further studies are needed to corroborate our findings in animal models that fully recapitulate the fatal human CHIKV infection.

In conclusion, our data provide a more comprehensive context for understanding the pathophysiological determinants of CHIK deaths, including viral and host factors. Also, our findings provide mechanistic insights that may be involved in CNS disease described during some CHIKV infections. Moreover, this work expands our knowledge of CHIK pathophysiology, which may enable future translational efforts at developing effective treatments, prognostic biomarkers, and strategies for clinical management.

## STAR★Methods

### Key resources table

**Table:** 

REAGENT or RESOURCE	SOURCE	IDENTIFIER
**Antibodies**		

anti-ZO1 antibody	Abcam	Cat# Ab221547; RRID: AB_2892660
anti-CD31/PECAM-1 antibody	Novus	Cat# NB1002284; RRID: AB_10002513
anti-CHIKV E2 antibody	Thermo Fisher Scientific	Cat# MA5-18180; RRID:AB_2539554
AlexaFluor488-conjugated anti-rabbit IgG	Thermo Fisher Scientific	Cat# A32731; RRID: AB_2633280
AlexaFluor 568-conjugated goat anti-mouse IgG	Thermo Fisher Scientific	Cat# A11031; RRID:AB_144696

**Bacterial and virus strains**		

Chikungunya virus strain S27	Universidade de São Paulo, Brazil	N/A
Chikungunya virus strain 181-25	University of Texas Medical Branch, USA	N/A
Chikungunya virus strain 1455/76	University of Texas Medical Branch, USA	N/A

**Biological samples**		

Sera, cerebrospinal fluid, and tissue samples from chikungunya patients in Brazil	Laboratório Central de Saúde Pública do Ceará; Fundação Oswaldo Cruz – Bahia; Universidade de São Paulo, Brazil	N/A
Serum samples from chikungunya patients and uninfected people in Panama	Gorgas Memorial Institute of Health Studies, Panama	N/A
Sera from healthy blood donors from Brazil	Universidade de São Paulo, Brazil	N/A

**Chemicals, peptides, and recombinant proteins**		

Triton X-100	Sigma-Aldrich	X100-500ML
DAPI (4',6-Diamidino-2-Phenylindole, Dihydrochloride)	Thermo Fisher Scientific	D1306
Triton X-100	Sigma-Aldrich	X100-500ML

**Critical commercial assays**		

QIAamp Viral RNA Mini Kit	QIAGEN	204443
NucleoSpin RNA Blood Mini kit	Macherey-Nagel	740200.5
TaqMan Fast Virus 1-Step Master Mix	Thermo Fisher Scientific	4444436
Human Magnetic Luminex Assay (LXSAHM) – 12 Plex	R&D Systems	LXSAHM
ProtoScript® II First Strand cDNA Synthesis Kit	New England Biolabs	E6560L
Q5 High-Fidelity DNA polymerase	New England Biolabs	M0491L
Agencourt AMPure XP	Beckman Coulter	A63880
Qubit dsDNA HS Assay Kit	QIAGEN	Q32851
Native Barcoding Expansion 96	Oxford Nanopore Technologies	SQK-NBD114.96
Ligation Sequencing Kit XL	Oxford Nanopore Technologies	SQK-LSK108D
R9.4.1 flowcell	Oxford Nanopore Technologies	FLO-MIN106D
NEBNext Ultra II Directional RNA Library Prep	New England Biolabs	E7775L
AMPure XP Beads	Beckman Coulter	A63882
Quick-RNA MagBead Kit	Zymo Research	R2133
ZyQuik sPECAM-1 ELISA kit	Thermo Fisher Scientific	99-0036

**Deposited data**		

Sequence alignments and analysis outputs	This paper	https://github.com/wmarciel/Chikungunya_pathophysiology
Chikungunya genomes	GenBank	PP336966-PP337021
Epidemiological data of chikungunya in Brazil	Github repository	https://github.com/wmarciel/Chikungunya_pathophysiology

**Experimental models: Cell lines**		

Vero E6 (kidney epithelial cells from non-human primates)	American Type Culture Collection	CRL-1586
Primary human brain microvascular endothelial cells	University of Texas Medical Branch, USA	N/A
Human primary astrocytes	University of Texas Medical Branch, USA	N/A

**Oligonucleotides**		

DENV-1f 5'-CAAAAGGAAGTCGYGCAATA-3'	Johnson et al.^25^	N/A
DENV-1r 5'-CTGAGTGAATTCTCTCTGCTR AAC-3'	Johnson et al.^25^	N/A
DENV-1p 5'- FAM-CATGTGGYTGGGAGC RCGC- BHQ1-3’	Johnson et al.^25^	N/A
DENV-2f 5'- CAGGCTATGGCACYGTCACGA T-3’	Johnson et al.^25^	N/A
DENV-2r 5'- CCATYTGCAGCARCACCATCTC-3’	Johnson et al.^25^	N/A
DENV-2p 5'- HEX- CTCYCCRAGAACGGGCCTC GACTTCAA - BHQ1-3’	Johnson et al.^25^	N/A
DENV-3f 5'- GGA CTR GAC ACA CGC ACC CA-3’	Johnson et al.^25^	N/A
DENV-3r 5'- CATGTCTCTACCTTCTCGACTTG YCT-3’	Johnson et al.^25^	N/A
DENV-3p 5'-RED- ACCTGGATGTCGGCTGAAG GAGCTTG- BHQ2-3’	Johnson et al.^25^	N/A
DENV-4f 5'-TTGTCCTAATGATGCTRG TCG-3’	Johnson et al.^25^	N/A
DENV-4r 5'- TCCACCYGAGACTCCTTCCA-3’	Johnson et al.^25^	N/A
DENV-4 p 5'-CY5- TYCCTACYCCTACGCATCG CATTCCG- BHQ2-3’	Johnson et al.^25^	N/A
CHIKV 874 5'-AAAGGGCAAACTCAGCTTCAC-3'	Lanciotti et al.^63^	N/A
CHIKV 961 5'-GCCTGGGCTCATCGTTATTC-3'	Lanciotti et al.^63^	N/A
CHIKV 899-FAM 5'CGCTGTGATACAGTGGTTT CGTGTG-3'	Lanciotti et al.^63^	N/A
ZIKV 1086 5′-CCGCTGCCCAACACAAG-3′	Lanciotti et al.^26^	N/A
ZIKV 1162c 5′-CCACTAACGTTCTTTTGCAGA CAT-3′	Lanciotti et al.^26^	N/A
ZIKV 1107-FAM 5′-AGCCTACCTTGACAAGC AGTCA GACACTCAA /3IABkFQ −3′	Lanciotti et al.^26^	N/A

**Software and algorithms**		

Luminex xPONENT software	Luminex Corporation	https://www.luminexcorp.com/xponent/#overview
Guppy version 4.4.1	Github repository	https://github.com/nanoporetech/pyguppyclient
SamTools v1.4	Li et al.^64^	https://samtools.sourceforge.net
Guppyplex	Github repository	https://github.com/artic-network/artic-ncov2019
Geneious Prime 2023.0.4	Geneious	https://www.geneious.com/download/
MAFFT	Katoh and Standley^65^	https://mafft.cbrc.jp/alignment/server/
IQ-TREE version 2	Minh et al.^66^	http://www.iqtree.org
BEAST	Suchard et al.^67^	http://beast.community
TempEst	Rambaut et al.^68^	http://beast.community/tempest
Tracer	Rambaut et al.^69^	http://beast.community/tracer
RDP4	Martin et al.^70^	http://web.cbio.uct.ac.za/∼darren/rdp.html
FigTree v1.4.4	GitHub repository	https://github.com/rambaut/figtree/releases
MetaboAnalyst 4.0	MetaboAnalyst	https://www.metaboanalyst.ca/
Progenesis QI Software	Waters	https://www.waters.com/waters/en_US/Progenesis-QI-Software/nav.htm?cid=134790655&locale=en_US
Cytoscape	National Institute of General Medical Sciences	https://cytoscape.org
R Statistical Computing Software	The R Foundation	https://www.r-project.org/
R-package tidyverse	Wickham et al.^71^	https://www.tidyverse.org
R-package tmap	Tennekes^72^	https://cran.r-project.org/web/packages/tmap/vignettes/tmap-getstarted.html
R-package ggbreak	GitHub repository	https://cran.r-project.org/web/packages/ggbreak/index.html
R-package ggpubr	GitHub repository	https://cran.r-project.org/web/packages/ggpubr/index.html
R-package	GitHub repository	https://cran.r-project.org/web/packages/scico/index.html
R-package sp	GitHub repository	https://cran.r-project.org/web/packages/sp/index.html

**Other**		

Genome Reference Consortium Human Build 3872	Genome Reference Consortium	https://www.ncbi.nlm.nih.gov/assembly/GCF_000001405.26/
Kyoto Encyclopedia of Genes and Genomes Pathway	Kanehisa et al.^30^	https://www.genome.jp/kegg/
Reactome Pathways Database	Gillespie et al.^31^	https://reactome.org/
UniProtKB/Swiss-Prot	UniProt Consortium et al.^73^	https://www.uniprot.org/uniprotkb?facets=reviewed%3Atrue&query=%2A
Metlin	Guijas et al.^74^	https://metlin.scripps.edu/

### Resource availability

All requests for information regarding reagents and resources should be directed to the lead contact and will be fulfilled by the lead contact or corresponding authors.

#### Lead contact

Further information and requests for resources and reagents should be directed to and will be fulfilled by Dr. William M. de Souza (wmdesouza@uky.edu).

#### Materials availability

This study did not generate new unique reagents.

#### Data and code availability

All statistical computing analyses were conducted using the RStudio.^75^ R packages necessary for analysis and visualization include tidyverse,^71^ ggbreak,^76^ ggpubr,^77^ scico,^78^ tmap,^72^ and sf.^79^ No custom code was developed. New sequences have been deposited in GenBank with accession numbers (PP336966-PP337021). The mass spectrometry metabolomic and Ct-value data is available on GitHub (https://github.com/wmarciel/Chikungunya_pathophysiology). The mass spectrometry proteomics data have been deposited to the ProteomeXchange Consortium via the PRIDE^80^ partner repository with the dataset identifier PXD043815 and 10.6019/PXD043815. Any additional information required to reanalyze the data reported in this paper is available from the lead contact upon request.

### Experimental models and subject details

#### Epidemiological dataset and analysis

National epidemiological data on suspected CHIK cases and deaths were obtained from the Brazilian Ministry of Health. This dataset included the aggregate number of annual suspected cases and deaths from CHIK per year from all states of Brazil from 2015 to 2023. Epidemiological analyses were performed in R studio (version 1.3.1073). The correlation between suspected CHIK-deaths per year and suspected CHIK cases per year in Brazil was determined by Pearson's correlation coefficients. The cumulative case-fatality ratio for the country and states was also calculated.

#### Sample collection, clinical information, histopathological analysis, and ethics statement

Sera, cerebrospinal fluid (CSF), and tissue samples were collected from a cohort of CHIK-deaths (n = 32) in Brazil, as previously reported.^12^ Briefly, these cases were investigated by the Central Public Health Laboratory and the Death Verification Service during the chikungunya outbreak in Ceará State in 2017. Additional blood samples were collected from patients with acute CHIK who survived (n = 39) during the CHIK epidemics between 2016 to 2019 in Brazil. Samples from blood donors and healthy individuals were included in the study, all of whom were negative for CHIKV RNA by RT-qPCR and non-reagent for IgM and IgG against CHIKV (n = 15). All residual samples were stored at -80°C for subsequent investigation. Tissue samples from CHIK-deaths were conserved in formalin-fixed blocks for histopathological analysis. Basic clinical and demographic data were collected through a questionnaire filled out by the patients or their relatives.

#### Cell culture and virus propagation

Vero E6 (ATCC, CRL-1586) cells were cultured in Dulbecco’s modified Eagle’s medium (Gibco, USA) supplemented with 5% fetal bovine serum (Gibco, USA) and 100 units/mL penicillin and streptomycin at 37°C with 5% CO_2_. Viral isolates of CHIKV strain 181-25 and strain 1455/76 (isolated from Bangkok, Thailand, in 1975) were propagated in Vero E6 cells and titrated by plaque assay.

#### Astrocytes and endothelial cells

Human cortical tissue was obtained as part of a protocol approved by the Albert Einstein College of Medicine, Rutgers University, and the University of Texas Medical Branch at Galveston. Astrocytes and endothelial cells were isolated, as described previously.^81^

Briefly, astrocytes were obtained from autologous human fetal cerebrum and released using 1× trypsin and collagenase (1 mg/ml). Endothelial cells were isolated from human umbilical veins using a collagenase solution (1 mg/ml) and then cultured on gelatin-coated plates using M199 media. After that, endothelial cells were seeded onto the underside of a gelatin-coated culture insert with 3-μm pores (1×10^5^ cells/insert). On the other hand, endothelial cells were seeded on the upperside of the tissue culture inserts (1.6×10^4^ cells/insert). The inserts were then immersed in tissue culture wells containing medium M199, 10% heat-inactivated newborn calf serum, and endothelial cell growth factor. Endothelial cells are grown to confluence for 72 hours, and the cultures are examined microscopically to assess monolayer integrity.

#### *In vitro* model of the blood-brain barrier

The blood-brain barrier (BBB) model was performed with a co-culture of human primary astrocytes and endothelial cells on opposite sides of gelatin-coated tissue culture inserts with 3 μm pores (BD-Falcon, USA) that permit astrocyte processes to penetrate the insert and establish contact with the endothelial cells as described previously.^—^^81^^,^^82^^,^^83^ In this model, endothelial cells differentiate and express BBB markers, such as the glucose transporter (GLUT-1) and γ-glutamyltranspeptidase, as a result of contact with astrocytes, and have enhanced expression of the tight junction proteins. Also, cocultures exhibit barrier resistance to ^3^H-inulin and albumin and did not present differences in transmigration properties of PBMC between these two types of cocultures.^82^^,^^84^

#### Protocol approval for human studies

This study was approved by the ethics committees from the University of Campinas, Brazil (#53910221.0.0000.5404). Procedures involving sPECAM-1 analysis were approved by the ethics committee from Gorgas Memorial Institute of Health Studies, Panama (#1166/CBI/ICGES/17).

### Method details

#### Real-time quantitative reverse transcription-polymerase chain reaction (RT-qPCR) for chikungunya, dengue, and Zika viruses

Tissue fragments of 1 cm^3^ from CHIK autopsies were homogenized using a mortar and pestle with 15 ml of Leibovitz's L-15 medium (Gibco, USA) supplemented with 10% Fetal Bovine Serum (FBS) (Gibco, USA) and 1% antibiotic-antimycotic (Gibco, USA). Then, the homogenized tissue samples were centrifuged at 1,500xg for 15 min at 4°C, and separated the supernatant and pellet were separated. Viral RNA was extracted from homogenized tissue and serum samples using the QIAamp Viral RNA Mini Kit (Qiagen, USA). Viral RNA was extracted from blood samples using the NucleoSpin RNA Blood Mini kit (Macherey-Nagel, Germany). CHIKV RNA was detected using RT-qPCR with probe and primer sets, as previously described (key resources table).^63^ The assays were performed with TaqMan™ Fast Virus 1-Step Master Mix (Applied Biosystems, USA) on the StepOnePlus™ Real-Time PCR System (Applied Biosystems, USA). CHIKV genome copy numbers were calculated using on a standard curve generated using RNA from an isolate of CHIKV strain S27. In addition, all samples were tested by RT-qPCR for ZIKV, and DENV using probes and primers sets as previously described (key resources table).^25^^,^^26^

#### Human cytokine immunoassay

The concentration of cytokines was determined using the Human Magnetic Luminex Assay (LXSAHM) – 12 Plex (R&D Systems, USA), according to the manufacturer’s instructions. The assay includes CXCL1/GRO alpha/KC/CINC-1, GM-CSF, IFN-alpha, IFN-beta, IFN-gamma, IL-1 beta/IL-1F2, IL-6, IL-10, IL-12 p70, IL-28A/IFN-λ2, IL-28B/IFN-λ3, and TNF-α. Briefly, magnetic beads were aliquoted in 96-well plates and washed. Serum samples from patients who died of CHIK (n = 13), patients with acute CHIK who survived (n = 12), and healthy blood donors (n = 15) were included in the assays. The 25μl serum samples were added to the plate containing the mixed antibody-linked beads, incubated at room temperature for 2 hours, then washed again, followed by incubation with biotin for 1 hour and a final 30 minutes of incubation in the presence of streptavidin-phycoerythrin. Results were obtained using the Bio-Plex 200 system (BioRad, USA) with Luminex xPONENT software (version 3.1), based on standard curves plotted through a 5-parameter logistic curve setting. The software allows extrapolating data from the curve based on the sensitivity and standard concentration range of detection. Performance validation of the assay is made by the manufacturer for each target analyte screened against all target analytes to confirm low antibody cross-reactivity. Then, statistical analyses were performed using the Wilcoxon signed-rank test with the Dunn-Bonferroni post-hoc test in the R studio version 1.3.1073.

#### Chikungunya virus genome sequencing and genome assembly

Samples positive for CHIKV RNA by RT-qPCR were submitted for genome sequencing using a targeted multiplex PCR scheme and the MinION platform (Oxford Nanopore Technologies, UK), as previously described.^85^ Briefly, the PCR products were cleaned using AmpureXP purification beads (Beckman Coulter, UK), and quantified using fluorimetry with the Qubit dsDNA High Sensitivity assay on the Qubit 3.0 instrument (Life Technologies, USA). After being normalized, the amplicons from each sample were pooled, barcoded, and processed using the Rapid Barcoding Kit 96 kit (EXP-NBD 196, Oxford Nanopore Technologies, UK). Following this, the sequencing libraries were generated using the SQK-LSK109 Kit (Oxford Nanopore Technologies, UK) and were loaded onto an R9·4·1 flow-cell (Oxford Nanopore Technologies, UK). Then, FAST5 files containing the raw signal data were base-called, demultiplexed, and trimmed using Guppy version 4.4.1 (Oxford Nanopore Technologies, UK). Subsequently, the reads were aligned against the CHIKV strain C302F/2016/BR (GenBank accession no. KY055011) using minimap2 version 2.17.r941^86^ and converted to a sorted BAM file using SAMtools.^64^ Next, length filtering, quality testing, primmer trimming, variant calling, and consensus sequences were performed for each barcode using guppyplex. CHIKV genome regions with a depth coverage below 20-fold were represented with “N” characters.

#### Chikungunya virus genomic dataset and phylogenetic analysis

A total of 46 new CHIKV genomic sequences with ≥75% coverage were generated. These were aligned with the 220 other Brazilian CHIKV-ECSA complete coding sequences available in the GenBank database as of June 10, 2023. Our multiple sequence alignment (MSA) was built using MAFFT version 7.450,^65^ and manual adjustment was conducted using Geneious Prime 2023.0.4. The dataset was screened for recombination events using all available methods in RDP version 4,^70^ but no evidence of recombination was found. A maximum likelihood (ML) phylogeny tree was performed using IQ-TREE version 2 under a GTR + I + γ model determined by ModelFinder.^87^^,^^88^ The ultrafast-bootstrap approach with 1,000 replicates was used to determine the statistical support for nodes for our ML phylogeny. Regressed root-to-tip genetic divergence was calculated against sampling dates to examine the temporal signal and identify sequences with low data quality of our datasets, such as assembly errors, sample contamination, data annotation errors, sequencing, and alignment errors.^68^ No obvious outliers were identified. The dated-phylogenetic tree was estimated using BEAST v.1.10.4 under a GTR + I + γ model,^67^ strict molecular clock model, and a Skygrid tree prior,^89^ and using BEAGLE to enhance computation speed.^90^ Lastly, the evolutionary analyses were run independently in duplicate for 50 million steps, sampling parameters and trees every 10,000 steps. Maximum clade credibility summary trees were generated using TreeAnnotator v.1.10.^69^

#### Metabolomics analysis

Metabolomic analysis was performed with serum samples from patients who died of CHIK (n = 12), patients with acute CHIK who survived (n = 12), and healthy blood donors (n = 15). Briefly, 20μl of each sample was added and mixed with 200μl of tetrahydrofuran and 780μl of methanol. The mixture was homogenized and centrifuged at 1,300xg, for 5 min, and at 4°C for the precipitation of denatured proteins. The supernatants contained metabolites of diverse chemical natures. Then, 5μl of the clear supernatant was diluted in 495μl of methanol, and samples were ionized by adding formic acid (0.1% final concentration) before mass spectrometric (MS) analysis. The sample was directly infused into HESI-Q-Exactive Orbitrap®-MS (Thermo Fisher Scientific, USA) equipment. Spectral data were acquired in 10 replicates per sample in the range of *m/z* 200-1700 on positive ion mode. MS parameters were set as follows: H_2_ sheath gas flow rate five units, capillary temperature 320°C, auxiliary gas heater temperature 33°C, spray voltage 3.70 kV, automatic gain control (AGC) at 1×10^6^, S-lens RF level 50, and injection time <2 ms. Relative intensities of *m/z* were compared between samples and groups using multivariate statistical analysis with a supervised PCA (Principal Component Analysis) and supervised PLS-DA (Partial Least Square - Discriminant Analysis) approaches available in MetaboAnalyst 4.0 online platform (https://www.metaboanalyst.ca/).^27^ Discriminant *m/z* features ranked by the VIP score underwent identification using Metlin (https://metlin.scripps.edu/) database for exact mass search accuracy ≤ 5 ppm.^74^ Selected markers were evaluated according to fold change (FC) variation and significance with a p-value < 0.01 (Wilcoxon test; two-sided). Markers normalized log-transformed intensity distribution is visualized through heatmaps and boxplots. A multiclass unsupervised PCA plot with annotated markers was used to visualize groups’ clustering.

#### Liquid chromatography–mass spectrometry proteomic analysis

Proteomic analysis was performed with serum samples from patients who died of CHIK (n = 13), patients with acute CHIK who survived (n = 11), and healthy blood donors (n = 15). First, the samples were treated with a protease inhibitor cocktail (Sigma-Aldrich, USA). Then, to remove the abundant plasma proteins, we depleted the 14 most plasma-abundant proteins using Pearson correlation and independent t-test with Benjamini-Hochberg adjusted p-value (FDR 5%) to correct for multiple comparisons as previously described elsewhere.^91^ Next, the total protein content was quantified using the BCA (bicinchoninic acid) method and diluted in buffer with Tris-HCL 100mM and 2M urea in a proportion of 1:1 v/v. Subsequently, we performed the Filter Aided Sample Preparation (FASP) method for tryptic digestion.^92^ Digested peptides from each sample were resuspended in 0.1% formic acid. The separation of tryptic peptides was performed on an ACQUITY MClass System (Waters Corporation, USA). Next, the separated peptides were analyzed by High Definition Synapt G2-Si Mass spectrometer directly coupled to the chromatographic system. Differential protein expression was evaluated with a data-independent acquisition (DIA) of shotgun proteomics analysis by Expression configuration mode (MSe). The mass spectrometer operated in “Expression Mode” switching between low (4 eV) and high (25–60 eV) collision energies on the gas cell, using a scan time of 1.0s per function over 50–2000 m/z. All spectra have been acquired in Ion Mobility Mode by applying a wave velocity for the ion separation of 1.000m/s and a transfer wave velocity of 175m/s. The processing of low and elevated energy, added to the data of the reference lock mass ([Glu1]-Fibrinopeptide B Standard) provides a time-aligned inventory of accurate mass retention time components for both the low and elevated-energy (exact mass retention time) each sample was run in three technical replicates. Continuum liquid chromatography–mass spectrometry (LC-MS) data from three replicate experiments for each sample were processed for qualitative and quantitative analysis using the software Progenesis (Waters Corporation, USA). The qualitative identification of proteins was obtained by searching the *Homo sapiens* database (UniProtKB/Swiss-Prot). Search parameters were set as automatic tolerance for precursor ions and for product ions, a minimum of 1 fragment ions matched per peptide, a minimum of three fragment ions matched per protein, a minimum of one unique peptide matched per protein, one missed cleavage, carbamidomethylation of cysteines fixed modification and oxidation of methionines as variable modifications, the false discovery rate of the identification algorithm < 1%. Thus, label-free quantitative analysis was obtained using the relative abundance intensity integrated into Progenesis QI software (Waters Corporation, USA), using all peptides identified for normalization. The expression analysis was performed considering technical replicates available for each experimental condition following the hypothesis that each group is an independent variable. In this study, only proteins were presented in at least two of three technical replicates reported, and a statistical ANOVA cutoff of >0.05 was adopted. Protein Interaction Analysis and Pathways Enrichment Analysis were performed in Cytoscape (https://cytoscape.org/), and in silico analyses were performed in an R environment. For Heatmap, volcano plot and PCA analysis, it was used a homemade Python language open-source software OmicScope.^93^

#### Molecular dynamics of chikungunya virus and MXRA8

Simulation systems of the CHIKV envelope proteins bound to the matrix remodeling associated 8 (MXRA8) receptor were constructed based on the crystallographic structure.^32^ CHARMM-GUI^94^ was used to model missing residues amid the protein chains and solvate the system in a 172x172x172 nm water box, considering a salt concentration of 150 mM NaCl. The H++ server^95^ was used to adjust the protonation states of sidechains, considering a pH of 7.4. The structure of CHIVK-E2 proteins was also modified to mimic the mutant, replacing the arginine residues at the 242 position with histidine. Molecular dynamic simulations were conducted on these systems using AMBER software^96^ with CHARMM36^97^ force field. The structural optimization process consisted of seven minimization stages, employing a combination of steepest descent and conjugate gradient methods.^98^ In the initial stage, a constant volume simulation was carried out for 5 picoseconds, spanning from 0 to 10 K. Subsequently, from the 2^nd^ to the 9^th^ stage, we extended the simulations for 1 nanosecond each to gradually increase the temperature from 10 to 300 K. In the 10^th^ heating stage, the system reached and maintained a temperature of 300 K. During this stage, a 5-nanosecond molecular dynamics simulation was conducted to equilibrate the system's density at constant pressure (1 bar) while preserving the temperature at 300 K. The Shake algorithm^99^ was applied to all hydrogen bonds within the system. Simulation trajectories of 700 ns were generated and analyzed for both wild-type and mutant systems using the isothermal–isobaric ensemble (NPT) ensemble. Simulation trajectories of 700 ns were generated and analyzed for both wild-type and mutant systems using the NPT ensemble. The lengths of the simulations were sufficient to ensure stabilization of the Root Mean Square Deviation (RMSD) for both native and R242H mutants (Figures S8 and S9). The binding free-energy was estimated using the Generalized Born model augmented with the hydrophobic solvent accessible surface area applied to molecular mechanics (MM/GBSA).^100^^,^^101^ All calculations were performed with the MolAICal software.^102^

#### Alanine-scanning mutagenesis

To identify potentially important residues for protein folding, we performed a computational alanine-scanning mutagenesis,^102^^,^^103^ consisting in systematic mutations of protein residues to alanine, ultimately measuring how the system stability is affected after deleting a specific amino acid side chain. The Fold-X suite was used to measure the free-energy differences upon mutation.^104^

#### Endothelial cell adhesion protein analysis

Primary human brain microvascular endothelial cells (BMVEC) were cultured on 35 mm cell culture plates coated with 0.5% gelatin (Cat no. G8-500, Thermo Fisher Scientific, USA) and infected with CHIKV as described above. After 24 hours, the cells were fixed with 4% paraformaldehyde in phosphate-buffered saline (PBS) for 20 min, washed in PBS twice, and permeabilized with 0.1% Triton X-100 (Sigma-Aldrich, USA) for 1 minute. Cells were washed twice with PBS, prior to blocking overnight at 4°C in super blocking solution (50mM EDTA, 0.1% gelatin from fish, 1% BSA, 0.1% horse serum). Cells were incubated with anti-ZO1 (Cat No. Ab221547, Abcam, USA) or anti-CD31/PECAM-1 antibody (Cat No., NB1002284, Novus, USA) and anti-CHIKV E2 (Cat no. MA5-18180, Thermo Fisher Scientific, USA), diluted in super blocking solution overnight at 4°C. After washing ten times in PBS, cells were incubated with an AlexaFluor488-conjugated anti-rabbit IgG (Cat no. A32731, Thermo Fisher Scientific, USA) to ZO-1 or PECAM staining and with an AlexaFluor 568-conjugated goat anti-mouse IgG (Cat no. A11031, Thermo Fisher Scientific, USA) for viral staining for two hours at room temperature. Then, the cells were washed thrice in PBS and incubated with 4′,6-diamidino-2-phenylindole (DAPI) for 5 min. Cells were washed once again, as before, prior to mounting within prolonged gold antifade reagent (Life Technologies, USA) and imaged on a Nikon A1 confocal microscope with spectral detection.

#### Isolation and viral infection of peripheral blood mononuclear cells

Primary human peripheral blood mononuclear cells (PBMC) from anonymous healthy donors were isolated from leukapheresis samples using a Ficoll-Paque density gradient (Sigma-Aldrich, USA) followed by fluorescence-activated cell sorting (FACS) to isolate lymphocytes and monocytes. Then, lymphocytes and monocytes were activated with phytohemagglutinin (PHA) (5 μg/ml) plus interleukin 2 (IL-2; 5%) in Roswell Park Memorial Institute (RPMI) 1640 for 48 hours in polypropylene tubes at a density of 2 × 10^6^ cells/mL. Monocytes were not differentiated into macrophages because they cannot adhere to polypropylene tubes. Next, cells were infected with CHIKV strain 1455/76 at an MOI of 0.1 infectious units/cell per 2 hours, washed, resuspended in fresh medium, and maintained in polypropylene tubes for an additional 3 days. Simultaneously, uninfected cells were activated with PHA plus IL-2 for 48 hours, washed, and maintained in polypropylene tubes in fresh media for an additional 3 days without any virus.

#### Assay of PBMC transmigration across the human blood-brain barrier model

Chikungunya virus-infected or uninfected PBMCs (3 × 10^5^ cells/well) were added to the top of each tissue culture insert. CCL-2 (100 ng/ml), or medium alone was added to the bottom chamber. After 24 hours, the number of PBMCs that transmigrated into the bottom chamber was analyzed by FACS using premixed human CD45, CD14, and CD16 monoclonal antibodies conjugated to FITC and phycoerythrin (PE), respectively, as described by instructions of the manufacturer (Caltag Laboratories, USA).^81^^,^^82^^,^^84^

#### Permeability analysis of the blood-brain barrier model

After 24 hours of uninfected or infected PBMC transmigration across the BBB model, the integrity of the barrier was determined by a permeability assay. Inserts were washed with phenol red-free DMEM and placed into 24-well tissue culture plates containing 400μl of phenol red-free DMEM with 10% FBS in each well. Albumin (0.45%) conjugated to Evans blue dye was used for transmigration experiments, while Dextran Alexa Fluor 680 (Cat no. D34680, Thermo Fisher Scientific, USA) or Fluorescein isothiocyanate–dextran 4kDa (Cat no. 46944, Thermo Fisher Scientific, USA) were used for permeability experiments with virus. The albumin or dextran (Alexa Fluor 680 or FITC-dextran) was added to the top of the insert, and after 30 min of incubation at 37°C, media were collected from the lower chamber and read spectrophotometrically at 620 nm for albumin or by fluorescence for FITC-dextran or Dextran Alexa Fluor 680 through the human BBB model. We determine the maximal optical density (OD) reading that could be obtained with this preparation using ethylenediaminetetraacetic acid (EDTA) as a measure of total BBB disruption. This value was set as that obtained after complete disruption of permeability, signifying the passage of all Evans blue dye-conjugated albumin through cocultures.

#### Quantification of the soluble form of platelet endothelial cell adhesion molecule-1

We measured levels of the soluble form of platelet endothelial cell adhesion molecule-1 (sPECAM-1) in media obtained from experiments of PBMC transmigration across the human BBB model using the ZyQuik sPECAM-1 ELISA kit, according to the protocol suggested by the manufacturer (Cat no. 99-0036, Thermo Fisher Scientific, USA) following the protocol suggested by the manufacturer. Additionally, we quantified the sPECAM-1 from serum obtained from the eight patients infected with CHIKV with less than 5 days of symptoms (acute CHIK phase) and eight CHIKV- and DENV-uninfected people, both determined by RT-qPCR^63^^,^^105^ at the Department of Research in Virology and Biotechnology at the Gorgas Memorial institute for Health Studies. The human samples also were quantified by ZyQuik sPECAM-1 ELISA kit. The limit of detection for the sPECAM-1 and sICAM-1 assays was 0.1 ng/ml.

### Quantification and Statistical Analysis

Several statistical methods were conducted to assess differences, correlations, and associations between groups. In the epidemiological analysis (Figure 1), the number of suspected chikungunya deaths over time was shown in a time series, along with the cumulative case-fatality ratio of chikungunya deaths per state presented as a map. Pearson's correlation coefficients were used to determine the correlation between suspected chikungunya deaths per year and suspected chikungunya cases in Brazil. The analyses of demographic and viral load data (Figure 2) include some methods, such as Welch's t-test was used to calculate the interval in days between symptom onset and sample collection (fatalities, n=30, survivors, n=35). The age distribution of chikungunya patients and control was determined using the Wilcoxon signed-rank test with the Dunn-Bonferroni posthoc test. Spearman's rank correlation coefficient was used to calculate the correlation between viremia and time from symptom onset to sample collection samples in days (fatalities, n=6, survivors, n=36). The cytokine responses and metabolomic signatures were analyzed using Wilcoxon signed-rank test with the Dunn-Bonferroni posthoc test and Wilcoxon test, respectively (Figure 3), for fatalities (n=13), survivors (n=12), and healthy controls (n = 15). The proteomic profiles (Figure 4) were evaluated using Pearson correlation coefficient distance, principal component analysis, and volcano plot displaying the log_2_-fold change against the t-test-derived -log_10_ statistical P (fatalities, n=13, and survivors, n=11, healthy controls, n=15). Lastly, statistical analyses of in vitro experiments were performed using the paired t-test, one-way ANOVA, and Kruskal-Wallis Test, as indicated in legend Figures 6 and 7.

## Supplemental information

Document S1. Figures S1–S9 and Tables S1 and S2

Document S2. Article plus supplemental information
