# Supplementary material for: Pathophysiology of chikungunya virus infection associated with fatal outcomes
Source: Cell Host Microbe. 2024 Apr 10;32(4):606–622.e8. doi: 10.1016/j.chom.2024.02.011 (PMC11018361; doi:10.1016/j.chom.2024.02.011)
Supplement: Document S1. Figures S1–S9 and Tables S1 and S2 [file mmc1.pdf]

Cell Host & Microbe, Volume *32*

Supplemental information

Pathophysiology of chikungunya virus infection associated with fatal outcomes

William M. de Souza, Marcilio J. Fumagalli, Shirlene T.S. de Lima, Pierina L. Parise, Deyse C.M. Carvalho, Cristian Hernandez, Ronaldo de Jesus, Jeany Delafiori, Darlan S. Candido, Victor C. Carregari, Stefanie P. Muraro, Gabriela F. Souza, Leda M. Simões Mello, Ingra M. Claro, Yamilka Díaz, Rodrigo B. Kato, Lucas N. Trentin, Clauber H.S. Costa, Ana Carolina B.M. Maximo, Karene F. Cavalcante, Tayna S. Fiuza, Vânia A.F. Viana, Maria Elisabeth L. Melo, Clarissa P.M. Ferraz, Débora B. Silva, Larissa M.F. Duarte, Priscilla P. Barbosa, Mariene R. Amorim, Carla C. Judice, Daniel A. Toledo-Teixeira, Mariana S. Ramundo, Patricia V. Aguilar, Emerson L.L. Araújo, Fabio T.M. Costa, Thiago Cerqueira-Silva, Ricardo Khouri, Viviane S. Boaventura, Luiz Tadeu M. Figueiredo, Rong Fang, Brechla Moreno, Sandra López-Vergès, Liana Perdigão Mello, Munir S. Skaf, Rodrigo R. Catharino, Fabiana Granja, Daniel Martins-de-Souza, Jessica A. Plante, Kenneth S. Plante, Ester C. Sabino, Michael S. Diamond, Eliseo Eugenin, José Luiz Proença-Módena, Nuno R. Faria, and Scott C. Weaver

Supplemental Figures

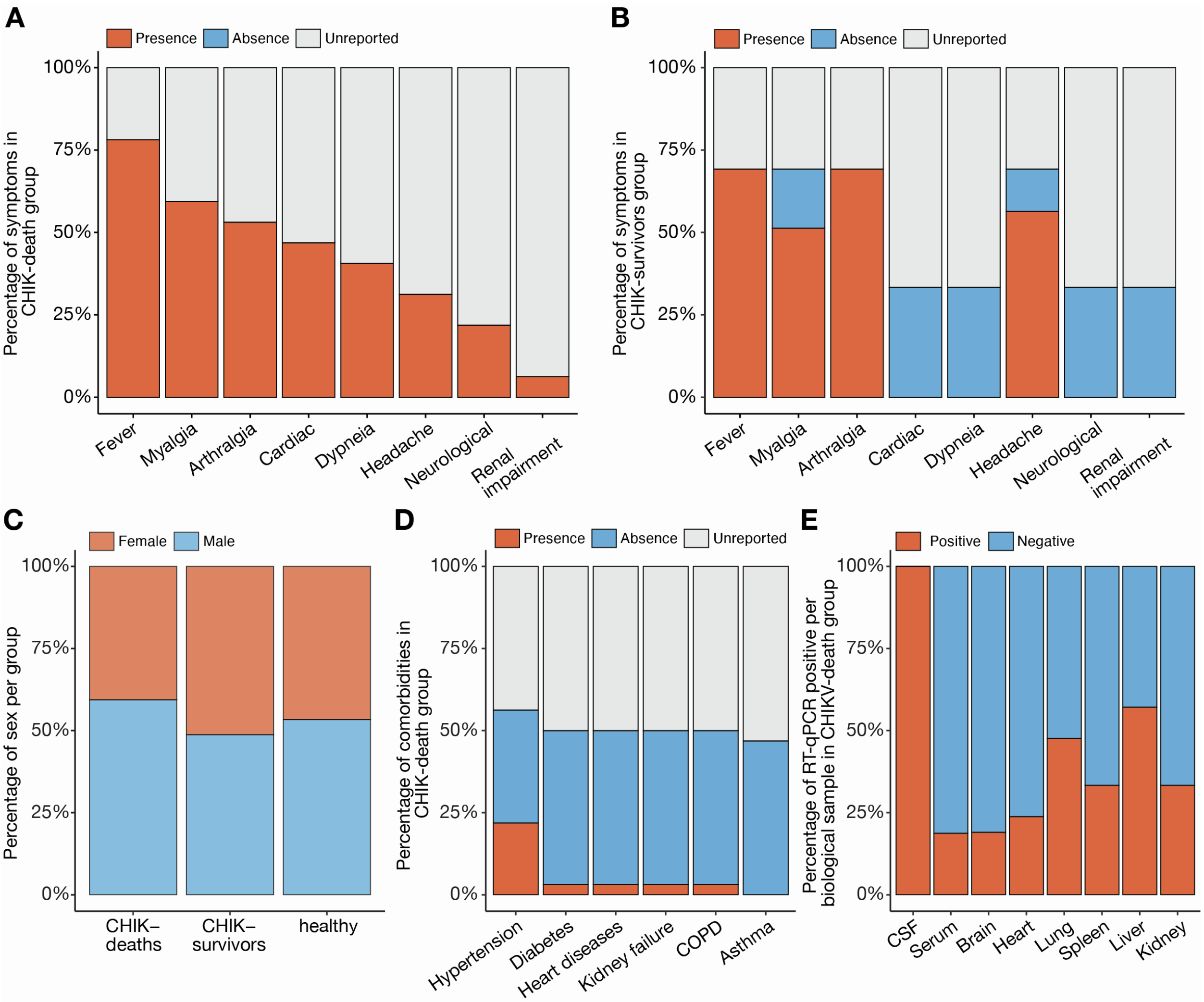

Figure S1. Clinical features of chikungunya patients, related to Figure 2. A) Symptoms and signs of fatal chikungunya cases. **B**) Symptoms and signs of chikungunya survivor cases. **C**) Percentage of females and males per group (CHIK-deaths, CHIK-survivors, and healthy blood donors. **D**) Comorbidities among fatal chikungunya cases. **E**) Percentage of RT-qPCR positive per biological sample in fatal chikungunya cases. CHIK, chikungunya. PFU, plaque-forming units. CSF, cerebrospinal fluid. RT-qPCR, Reverse transcription-quantitative polymerase chain reaction. COPD, Chronic obstructive pulmonary disease.

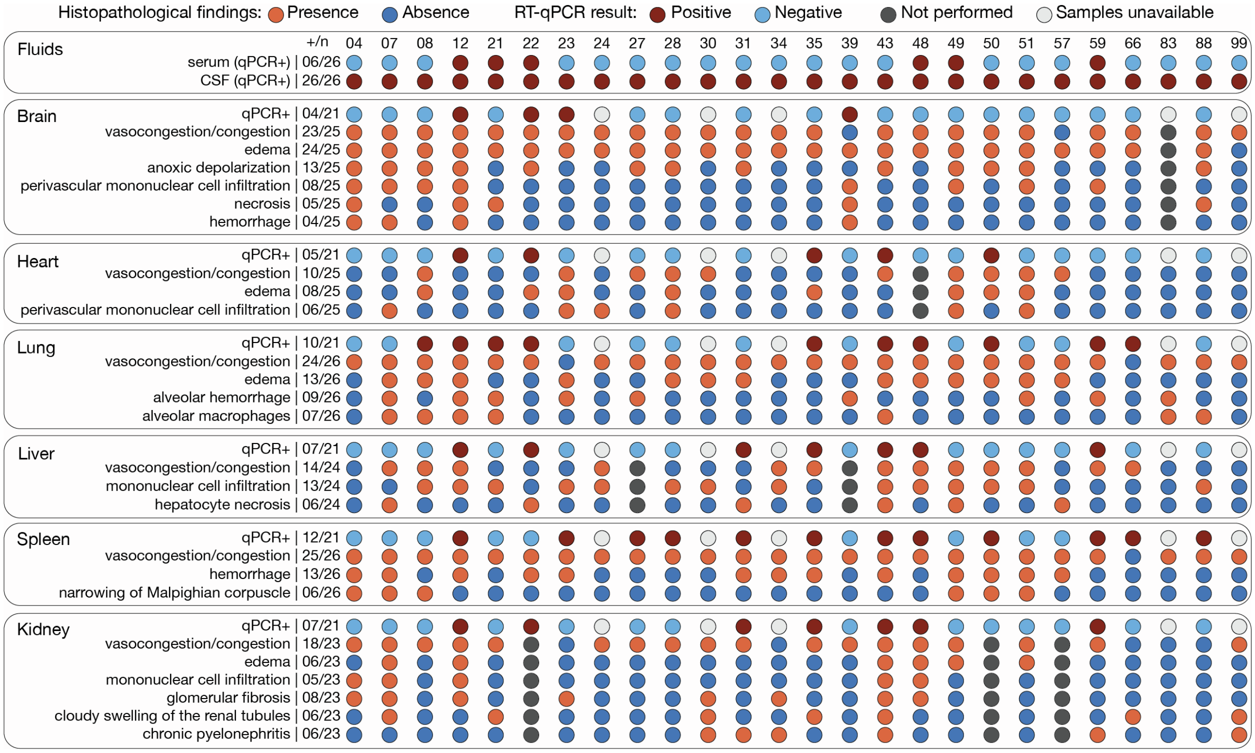

Figure S2. Histopathology findings from the necropsy of CHIK-deaths cases (n = 26), related to Figure 2. CHIK, chikungunya. PFU, plaque-forming units. CSF, cerebrospinal fluid. RT-qPCR, Reverse transcription-quantitative polymerase chain reaction. qPCR+, sample positive by RT-qPCR.

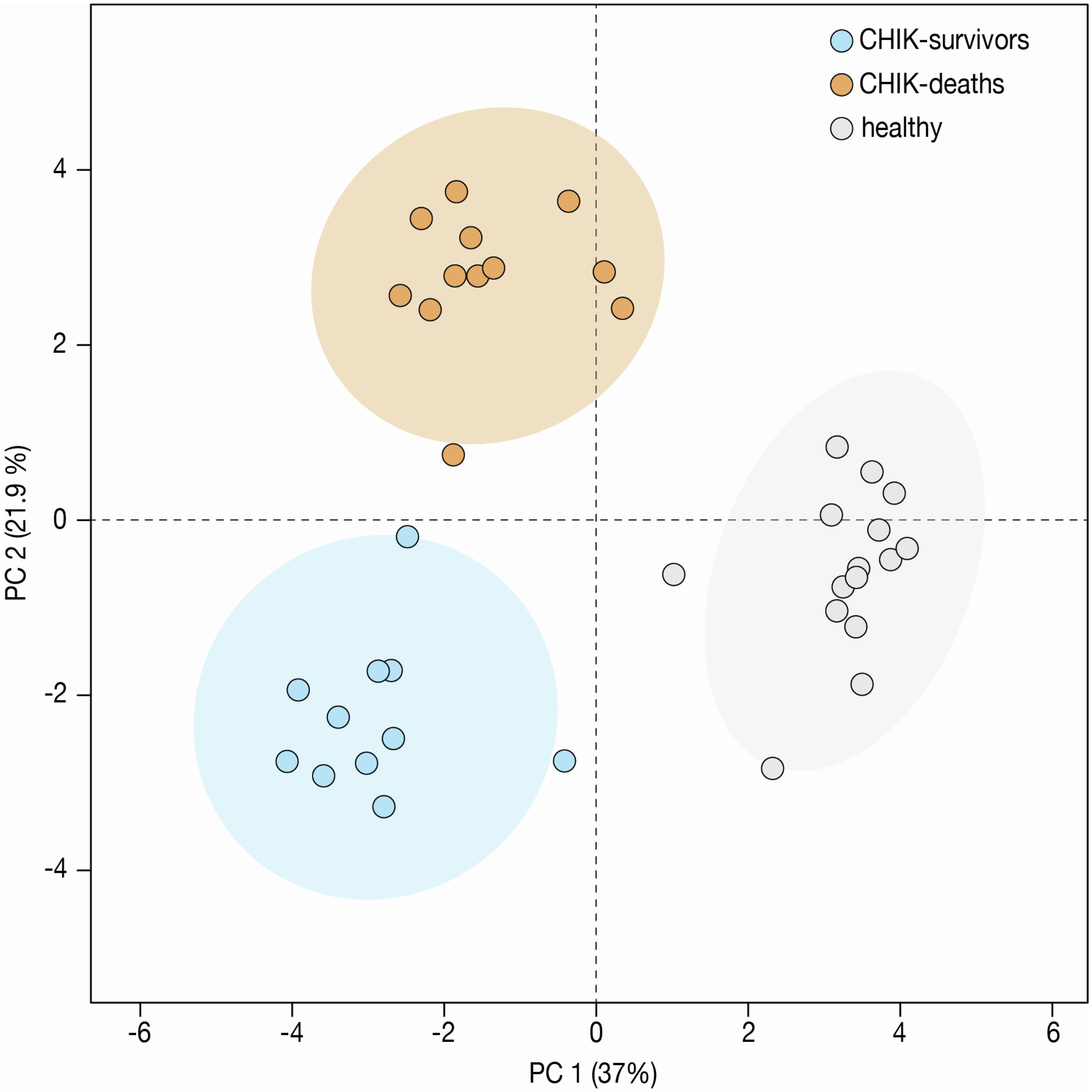

Figure S3. Principal component analysis of the metabolomics data from the serum samples of chikungunya patients (i.e., deaths and survivors) compared with healthy blood donors, related to Figure 3. Each dot represents one patient sample, color-coded for the different groups as described in the figure.

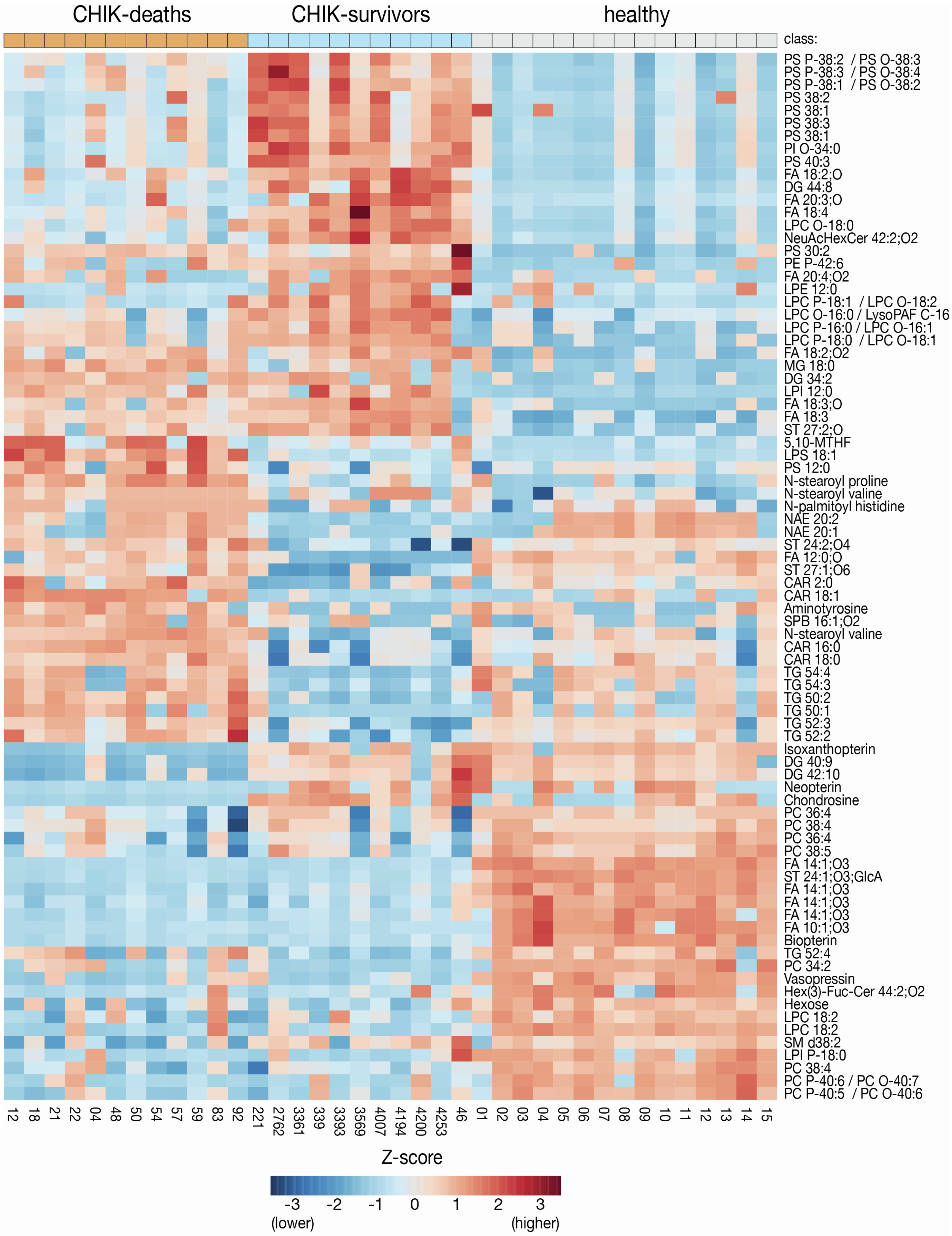

Figure S4. Heatmap analysis of selected metabolomic markers for chikungunya fever patients (i.e., fatal and survivors) compared with blood donors (healthy), related to Figure 3. Normalized log-transformed ion intensities (Z-score) are scaled from blue (low intensity) to red (high intensity). Samples

were clustered according to Pearson correlation coefficient distance. FA, fatty acid. CAR, acylcarnitine. LPC, lysophosphatidylcholine. LPE, lysophosphatidylethanolamine. LPI, lysophosphatidylinositol. LPS, lysophosphatidylserine. PC, phosphatidylcholine. PE, phosphatidylethanolamine. PI, phosphatidylinositol. PS, phosphatidylserine. DG, diacylglycerol. MG, monoacylglycerol. TG, triacylglycerol. Cer, ceramide. Fuc, fucosyl. Hex, hexose. NeuAcHexCer, ganglioside. NAE, anandamide. SM, sphingomyelin. SPB, sphingosine. ST, sterol lipids. GLcA, glucuronide. 5-MTHF, 5-methyltetrahydrofolate.

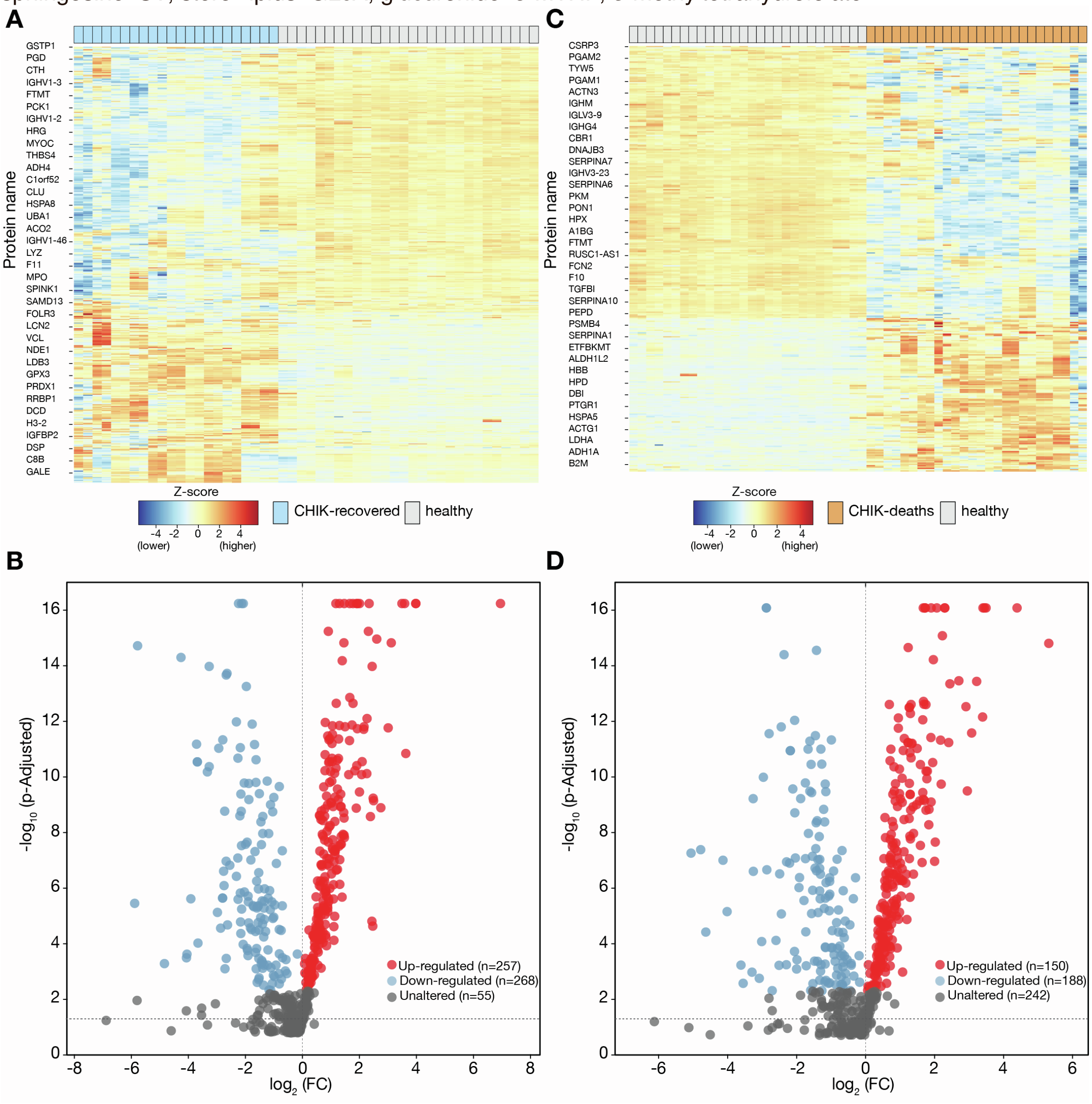

Figure S5. Proteomics signatures of chikungunya patients compared to healthy controls, related to Figure 4. The serum proteomic profiles of chikungunya patients (deaths, n=13, and survivors, n=11) were compared with healthy controls (n=15). **A)** Heatmap analysis of selected proteomic markers for chikungunya survivors compared with blood donors (healthy) shows the relativity intensities. The relative intensities (Z-score) are scaled from blue (low intensity) to red (high intensity). Samples were clustered according to Pearson correlation coefficient distance. **B)** Volcano plot displaying the $\log_2$-fold change (x-axis) against the t-test–derived $-\log_{10}$ statistical P value (y-axis) for all proteins differentially expressed

between chikungunya survivors and blood donors (healthy). Proteins with significantly decreased levels ($p < 0.05$) are shown in blue, and proteins with significantly increased levels are noted in red. The gray circles indicate proteins non-regulated. All proteins are shown. **C)** Heatmap analysis of selected proteomic markers for chikungunya deaths compared with blood donors (healthy) shows the Log_2 FC. The relative intensities (Z-score) are scaled from blue (low intensity) to red (high intensity). Samples were clustered according to Pearson correlation coefficient distance. **D)** Volcano plot displaying the log_2-fold change (x-axis) against the t-test–derived $-log_{10}$ statistical P-value (y-axis) for all proteins differentially expressed between chikungunya deaths and blood donors (healthy). Proteins with significantly decreased levels ($p < 0.05$) are shown in blue, and proteins with significantly increased levels are noted in red. The gray circles indicate proteins non-regulated. All of the proteins are shown. Gene symbols (abbreviation names) for protein-coding genes followed the nomenclature available on HUGO Gene Nomenclature Committee.

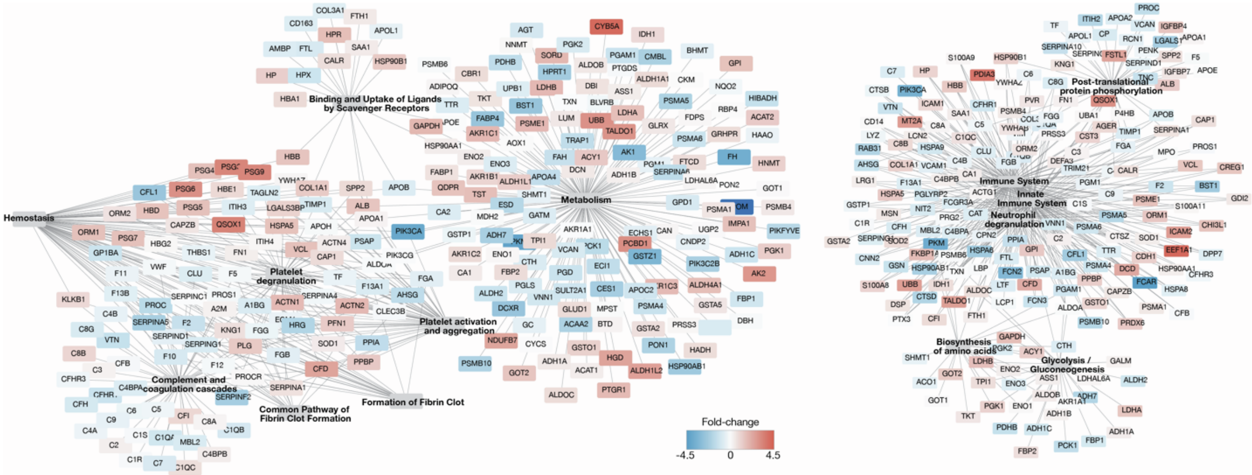

Figure S6. Protein–protein interaction analysis from chikungunya survivors compared to healthy blood donors, related to Figure 4. Gene symbols (abbreviation names) for protein-coding genes followed the nomenclature approved by the HUGO Gene Nomenclature Committee.

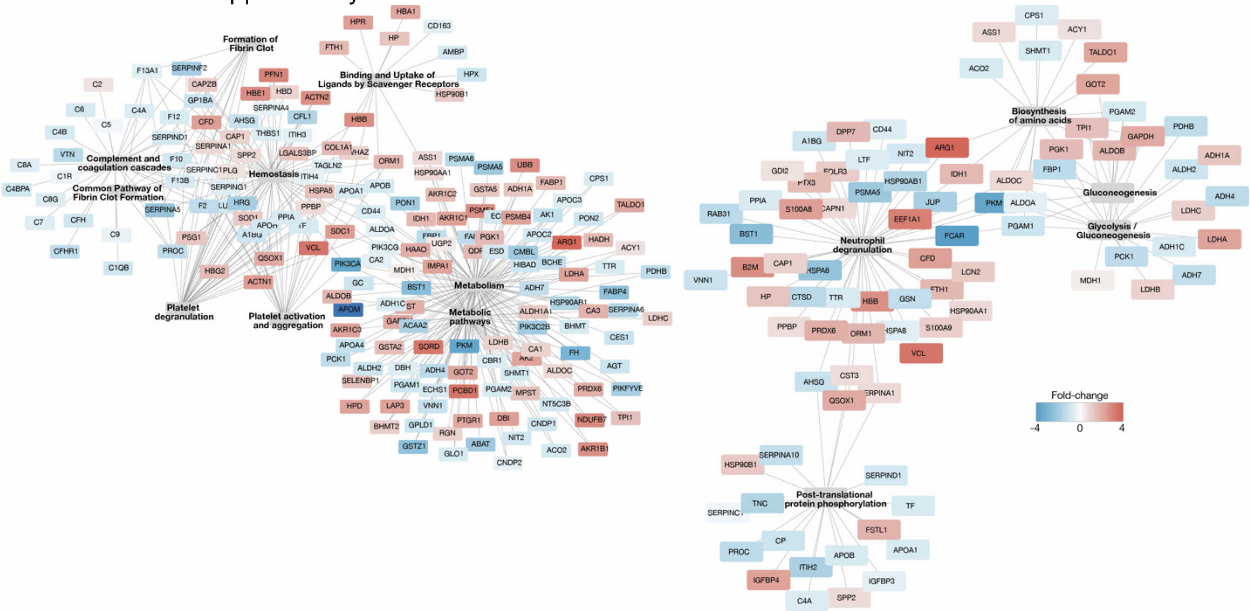

Figure S7. Protein–protein interaction analysis from chikungunya deaths compared to healthy blood donors, related to Figure 4. Gene symbols (abbreviation names) for protein-coding genes followed the nomenclature approved by the HUGO Gene Nomenclature Committee.

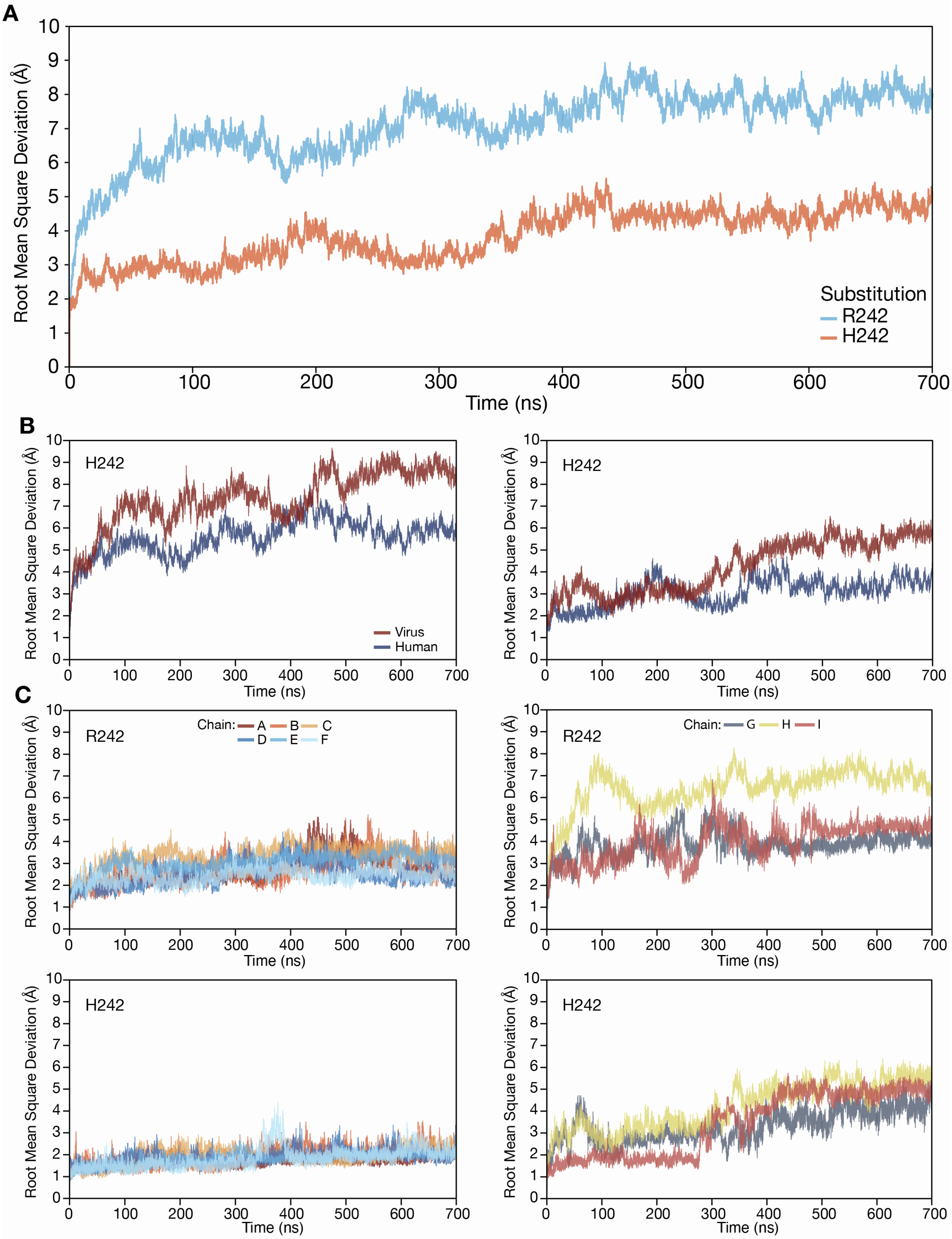

Figure S8. Molecular dynamic stimulations of R242H substitutions in envelope 2 protein of chikungunya virus with the Matrix Remodeling Associated 8 (MXRA8) receptor, related to Figure 5.

A) Root mean square deviation (RMSD) for the ancestral E2-R242 (blue line) and the E2-H242 (orange line) substitution with respect to their E2-E3 proteins and the hMXRA8 receptor during 700 nanoseconds of molecular dynamic simulations. **B)** RMSD for the substitution systems E2-R242 (left) and E2-H242 (right) with respect to their viral proteins (blue lines) and the hMXRA8 receptor (red lines) during 700 nanoseconds of molecular dynamic simulations. **C)** RMSD for the ancestral and derived residues E2-R242 and E2-H242 with respect to their individual viral chain proteins and the human receptor during 700 ns of molecular dynamic simulation.

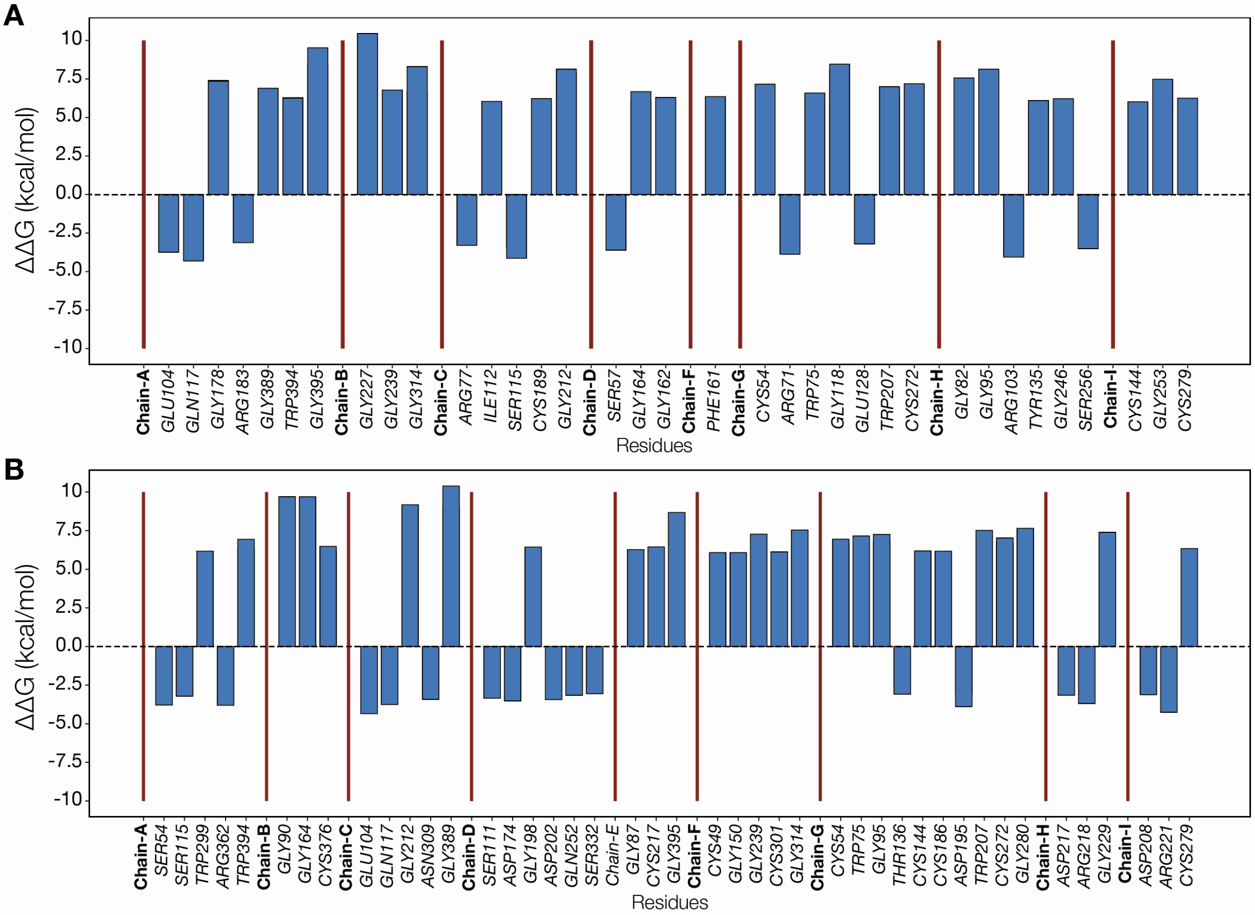

Figure S9. Folding energy differences after alanine-scanning mutagenesis of key residues in E2-H242 and E2-R242 substitutions of chikungunya virus, related to Figure 5 and STAR Methods. A) Chikungunya virus with envelope 2 with arginine (R) in position 242. **B)** Chikungunya virus with envelope 2 with histidine (H) in position 242. Residues were selected based on an energy threshold of >5.0 kcal/mol or <2.5 kcal/mol. Chains were named according to the Protein Data Bank (https://www.rcsb.org/).

Table Supplementary 1. Results from RT-qPCR and cytokine profile from chikungunya fatal cases, related to Figures 2 and 3.

RT-qPCR					Cytokines							
	CSF		Blood		Serum (pg/ml)							
ID	Ct	PFU[1]	Ct	PFU[1]	IL-6	IL-8	IFN-α	TNF-α	CCL-2	IL-10	INF-λ3	GM-CSF
1	30	1.24	-	-	n.t.	n.t.	n.t.	n.t.	n.t.	n.t.	n.t.	n.t.
4	29.12	2.38	-	-	363.12	110.71	250.48	2.77	114.05	low	114.05	2.79
7	33.97	0.07	-	-	77746.3	low	7.48	503.06	227.89	1444.44	227.89	73.43
8	29.06	2.49	-	-	n.t.	n.t.	n.t.	n.t.	n.t.	n.t.	n.t.	n.t.
12	23.73	128.93	24.61	65.43	1342.09	113.54	0.98	182.15	79.1	low	79.1	2.26
18	32.76	0.16	-	-	2494.67	85.6	0.98	0.84	114.05	1.31	114.05	2.26
21	32.7	0.17	32.54	0.17	39512.6	3245.95	7.79	1191.47	160.12	85.19	160.12	344.81
22	25.61	32.03	24.76	57.9	931.29	138.96	0.73	7.85	130.05	low	130.05	1.73
23	37.02	0.01	-	-	n.t.	n.t.	n.t.	n.t.	n.t.	n.t.	n.t.	n.t.
24	34.27	0.05	-	-	n.t.	n.t.	n.t.	n.t.	n.t.	n.t.	n.t.	n.t.
25	33.99	0.06	-	-	n.t.	n.t.	n.t.	n.t.	n.t.	n.t.	n.t.	n.t.
27	33.89	0.07	-	-	n.t.	n.t.	n.t.	n.t.	n.t.	n.t.	n.t.	n.t.
28	36.69	0.01	-	-	n.t.	n.t.	n.t.	n.t.	n.t.	n.t.	n.t.	n.t.
30	27.57	7.5	-	-	n.t.	n.t.	n.t.	n.t.	n.t.	n.t.	n.t.	n.t.
31	27.95	5.66	-	-	n.t.	n.t.	n.t.	n.t.	n.t.	n.t.	n.t.	n.t.
34	28.06	5.22	-	-	n.t.	n.t.	n.t.	n.t.	n.t.	n.t.	n.t.	n.t.
35	34.45	0.05	-	-	n.t.	n.t.	n.t.	n.t.	n.t.	n.t.	n.t.	n.t.
39	27.54	7.67	-	-	n.t.	n.t.	n.t.	n.t.	n.t.	n.t.	n.t.	n.t.
43	26.14	21.63	-	-	n.t.	n.t.	n.t.	n.t.	n.t.	n.t.	n.t.	n.t.
48	33.95	0.07	25.38	36.55	69380.9	low	330.3	209.85	160.12	30.82	160.12	6.76
49	34.66	0.04	32.76	0.14	n.t.	n.t.	n.t.	n.t.	n.t.	n.t.	n.t.	n.t.
50	37.12	0.01	-	-	35875.8	18041.5	5.5	14.37	160.12	117.28	160.12	7.94
51	35.67	0.02	-	-	n.t.	n.t.	n.t.	n.t.	n.t.	n.t.	n.t.	n.t.
54	29.25	2.16	-	-	7235.66	705.92	2.59	13.96	114.05	low	114.05	3.89
57	29.18	2.28	-	-	1444.12	134.22	1.77	22.53	79.1	14.15	79.1	4.45
59	21.88	507.57	22.94	229.08	64342.3	low	16.84	48.42	145.37	12.03	145.37	340.88
66	35.49	0.02	-	-	n.t.	n.t.	n.t.	n.t.	n.t.	n.t.	n.t.	n.t.
99	35.77	0.02	-	-	n.t.	n.t.	n.t.	n.t.	n.t.	n.t.	n.t.	n.t.
83	35.05	0.03	-	-	2166.76	823.91	2.04	2.77	79.1	low	79.1	1.73
88	32.2	0.24	-	-	n.t.	n.t.	n.t.	n.t.	n.t.	n.t.	n.t.	n.t.
91	36.9	0.01	-	-	n.t.	n.t.	n.t.	n.t.	n.t.	n.t.	n.t.	n.t.
92	31.1	0.55	-	-	6.29	198.12	1.37	1.14	97.16	low	97.16	low

Legend: ID, patient identification. Ct, cycle threshold value. PFU, plaque-forming units equivalents/ml. pg, picogram. ml, mililiter. CSF, cerebrospinal fluid. Low, quantification below of limit of detection. n.t., not

tested. IL-6, interleukin 6. IL-8, interleukin 8. IL-10, interleukin 10. IFN-α, interferon alpha. TNF-α, Tumour necrosis factor alpha. CCL-2, chemokine ligand 2. INF-λ3, Interferon lambda 3. GM-CSF, Granulocyte-macrophage colony-stimulating factor, -, negative.

Table Supplementary 2. Information on chikungunya cases sequenced in this study, related to Figure 5 and STAR Methods.

ID	Outcome	Age - years	Sex	Sample type	Onset symptom	Sample collection	Municipality	State	Genbank No.
50B	death	83	F	Spleen	10-May-17	15-May-17	Fortaleza	Ceará	PP336966
35P	death	36	F	Lung	16-Apr-17	23-Apr-17	Fortaleza	Ceará	PP336967
35B	death	36	F	Spleen	16-Apr-17	23-Apr-17	Fortaleza	Ceará	PP336968
59P	death	1	M	Lung	23-May-17	30-May-17	Fortaleza	Ceará	PP336969
59B	death	1	M	Spleen	23-May-17	30-May-17	Fortaleza	Ceará	PP336970
59S	death	1	M	Blood	23-May-17	30-May-17	Fortaleza	Ceará	PP336971
21P	death	71	F	Lung	1-Apr-17	9-Apr-17	Fortaleza	Ceará	PP336972
12P	death	74	F	Lung	15-Mar-17	22-Mar-17	Fortaleza	Ceará	PP336973
12B	death	74	F	Spleen	15-Mar-17	22-Mar-17	Fortaleza	Ceará	PP336974
12S	death	74	F	Serum	15-Mar-17	22-Mar-17	Fortaleza	Ceará	PP336975
12R	death	74	F	Kidney	15-Mar-17	22-Mar-17	Fortaleza	Ceará	PP336976
12C	death	74	F	Hearth	15-Mar-17	22-Mar-17	Fortaleza	Ceará	PP336977
12L	death	74	F	CSF	15-Mar-17	22-Mar-17	Fortaleza	Ceará	PP336978
22P	death	84	M	Lung	10-Apr-17	16-Apr-17	Fortaleza	Ceará	PP336979
22B	death	84	M	Spleen	10-Apr-17	16-Apr-17	Fortaleza	Ceará	PP336980
22F	death	84	M	Liver	10-Apr-17	16-Apr-17	Fortaleza	Ceará	PP336981
22C	death	84	M	Hearth	10-Apr-17	16-Apr-17	Fortaleza	Ceará	PP336982
22L	death	84	M	CSF	10-Apr-17	16-Apr-17	Fortaleza	Ceará	PP336983
22S	death	84	M	Serum	10-Apr-17	16-Apr-17	Fortaleza	Ceará	PP336984
48S	death	82	M	Serum	10-May-17	15-May-17	Fortaleza	Ceará	PP336985
54L	death	31	M	CSF	19-May-17	25-May-17	Caucaia	Ceará	PP336986
43B	death	79	M	Spleen	28-Apr-17	8-May-17	Fortaleza	Ceará	PP336987
27B	death	22	M	Spleen	11-Apr-17	14-Apr-17	Caucaia	Ceará	PP336988
8L	death	36	F	CSF	-	19-Mar-17	Caucaia	Ceará	PP336989
4S	survived	66	M	Serum	4-Mar-17	6-Mar-17	Fortaleza	Ceará	PP336990
221-1S	survived	35	F	Serum	5-Jun-17	6-Jun-17	Itatira	Ceará	PP336991
ZK0162	survived	43	M	Serum	6-Apr-16	9-Apr-16	Campo Formoso	Bahia	PP336992
ZK0173	survived	22	M	Serum	26-Apr-16	28-Apr-16	Itabuna	Bahia	PP336993
105S	survived	24	M	Serum	28-May-19	30-May-19	Rio de Janeiro	Rio de Janeiro	PP336994
ZK0194	survived	28	M	Serum	25-Apr-16	28-Apr-16	Itabuna	Bahia	PP336995
ZK0196	survived	30	M	Serum	27-Apr-16	28-Apr-16	Itabuna	Bahia	PP336996
ZK0188	survived	43	F	Serum	27-Apr-16	28-Apr-16	Itabuna	Bahia	PP336997

19S	survived	33	M	Serum	23-Apr-19	26-Apr-19	Rio de Janeiro	Rio de Janeiro	PP336998
77S	survived	30	M	Serum	20-May-19	23-May-19	Rio de Janeiro	Rio de Janeiro	PP336999
196	survived	37	F	Serum	31-Jul-19	2-Aug-19	Rio de Janeiro	Rio de Janeiro	PP337000
ZK0088	survived	15	F	Serum	6-Apr-16	7-Apr-16	Campo Formoso	Bahia	PP337001
ZK0130	survived	18	M	Serum	6-Apr-16	8-Apr-16	Campo Formoso	Bahia	PP337002
ZK0143	survived	45	F	Serum	7-Apr-16	8-Apr-16	Campo Formoso	Bahia	PP337003
ZK0313	survived	57	F	Serum	10-Jun-17	13-Jun-17	-	Bahia	PP337004
90S	survived	36	F	Serum	28-May-19	31-May-19	Rio de Janeiro	Rio de Janeiro	PP337005
197S	survived	37	F	Serum	6-Aug-19	7-Aug-19	Rio de Janeiro	Rio de Janeiro	PP337006
16S	survived	60	M	Serum	24-Apr-19	25-Apr-19	Duque de Caxias	Rio de Janeiro	PP337007
21S	survived	60	F	Serum	25-Apr-19	26-Apr-19	Rio de Janeiro	Rio de Janeiro	PP337008
ZK0135	survived	53	M	Serum	7-Apr-16	8-Apr-16	Campo Formoso	Bahia	PP337009
ZK0170	survived	29	M	Serum	25-Apr-16	28-Apr-16	Itabuna	Bahia	PP337010
ZK0101	survived	48	F	Serum	4-Apr-16	8-Apr-16	Campo Formoso	Bahia	PP337011
CH14	survived	34	F	Serum	-	17-May-17	São Paulo	São Paulo	PP337012
CH16	survived	3	M	Serum	-	20-Apr-17	São Paulo	São Paulo	PP337013
CH21	survived	21	F	Serum	-	25-Aug-17	São Luís	Maranhão	PP337014
CH22	survived	38	F	Serum	-	29-Aug-17	São Luís	Maranhão	PP337015
CH23	survived	21	F	Serum	-	4-Sep-17	São Luís	Maranhão	PP337016
CH24	survived	34	F	Serum	-	16-May-17	São Luís	Maranhão	PP337017
CH25	survived	37	F	Serum	-	4-Jun-17	São Luís	Maranhão	PP337018
CH26	survived	23	F	Serum	-	2-Jun-17	São Luís	Maranhão	PP337019
CH42	survived	44	F	Serum	-	30-Apr-16	Maceió	Alagoas	PP337020
CH06	survived	21	F	Serum	-	12-Jul-18	Andradina	São Paulo	PP337021

Legend: F, female. M, male. CSF, cerebrospinal fluid.
